# Evaluating the impact of the Radiomics Quality Score: a systematic review and meta-analysis

**DOI:** 10.1007/s00330-024-11341-y

**Published:** 2025-01-10

**Authors:** Nathaniel Barry, Jake Kendrick, Kaylee Molin, Suning Li, Pejman Rowshanfarzad, Ghulam M. Hassan, Jason Dowling, Paul M. Parizel, Michael S. Hofman, Martin A. Ebert

**Affiliations:** 1 School of Physics, Mathematics and Computing, University of Western Australia, Crawley, WA Australia; 2 Centre for Advanced Technologies in Cancer Research (CATCR), Perth, WA Australia; 3 Australian Centre for Quantitative Imaging, Medical School, University of Western Australia, Crawley, WA Australia; 4 Department of Radiation Oncology, Sir Charles Gairdner Hospital, Nedlands, WA Australia; 5 The Australian e-Health Research Centre, CSIRO, Brisbane, QLD Australia; 6 David Hartley Chair of Radiology, Royal Perth Hospital and University of Western Australia, Perth, WA Australia; 7 Medical School, University of Western Australia, Perth, WA Australia; 8 Prostate Cancer Theranostics and Imaging Centre of Excellence (ProsTIC); Molecular Imaging and Therapeutic Nuclear Medicine, Cancer Imaging, Peter MacCallum Centre, Melbourne, VIC Australia; 9 Sir Peter MacCallum Department of Oncology, University of Melbourne, Melbourne, VIC Australia

**Keywords:** Radiomics, Radiomics Quality Score, Systematic review, Meta-analysis, Precision medicine

## Abstract

**Objectives:**

Conduct a systematic review and meta-analysis on the application of the Radiomics Quality Score (RQS).

**Materials and methods:**

A search was conducted from January 1, 2022, to December 31, 2023, for systematic reviews which implemented the RQS. Identification of articles prior to 2022 was via a previously published review. Quality scores of individual radiomics papers, their associated criteria scores, and these scores from all readers were extracted. Errors in the application of RQS criteria were noted and corrected. The RQS of radiomics papers were matched with the publication date, imaging modality, and country, where available.

**Results:**

A total of 130 systematic reviews were included, and individual quality scores 117/130 (90.0%), criteria scores 98/130 (75.4%), and multiple reader data 24/130 (18.5%) were extracted. 3258 quality scores were correlated with the radiomics study date of publication. Criteria scoring errors were discovered in 39/98 (39.8%) of articles. Overall mean RQS was 9.4 ± 6.4 (95% CI, 9.1–9.6) (26.1% ± 17.8% (25.3%–26.7%)). Quality scores were positively correlated with publication year (Pearson *R* = 0.32, *p* < 0.01) and significantly higher after publication of the RQS (year < 2018, 5.6 ± 6.1 (5.1–6.1); year ≥ 2018, 10.1 ± 6.1 (9.9–10.4); *p* < 0.01). Only 233/3258 (7.2%) scores were ≥ 50% of the maximum RQS. Quality scores were significantly different across imaging modalities (*p* < 0.01). Ten criteria were positively correlated with publication year, and one was negatively correlated.

**Conclusion:**

Radiomics study adherence to the RQS is increasing with time, although a vast majority of studies are developmental and rarely provide a high level of evidence to justify the clinical translation of proposed models.

**Key Points:**

***Question*** *What level of adherence to the Radiomics Quality Score have radiomics studies achieved to date, has it increased with time, and is it sufficient?*

***Findings*** *A meta-analysis of 3258 quality scores extracted from 130 review articles resulted in a mean score of 9.4* ± *6.4. Quality scores were positively correlated with time.*

***Clinical relevance*** *Although quality scores of radiomics studies have increased with time, many studies have not demonstrated sufficient evidence for clinical translation. As new appraisal tools emerge, the current role of the Radiomics Quality Score may change.*

## Introduction

Medical care is becoming increasingly data-driven as we shift into an era of precision medicine [1]. Given the vastness of the data collected, clinicians cannot be expected to promptly and adequately assess their relevance to patient treatment without appropriate tools [2]. Imaging data are a large contributor, with artificial intelligence levelling the playing field, ingesting Terabytes to Petabytes of data to develop models that can positively impact clinical workflows and trials [3–5].

Quantitatively rich medical imaging data is rarely utilised by radiologists and nuclear medicine physicians beyond one-dimensional measurements or semiquantitative analyses [6, 7]. However, textural analysis that spatially characterises regions/volumes of interest may provide insight into the underlying pathophysiology [8–11]. This high-throughput extraction of image features was coined “radiomics”; handcrafted features from radiographic images to serve as a bridge to genomic and proteomic expression [12, 13]. Its primary application is in predictive modelling; however, the rapid adoption and evolution of radiomics analysis has raised concerns about standardisation, workflow reproducibility, high dimensional feature spaces, model stability, and generalisability for clinical translation [14–22].

**Fig. 1:**
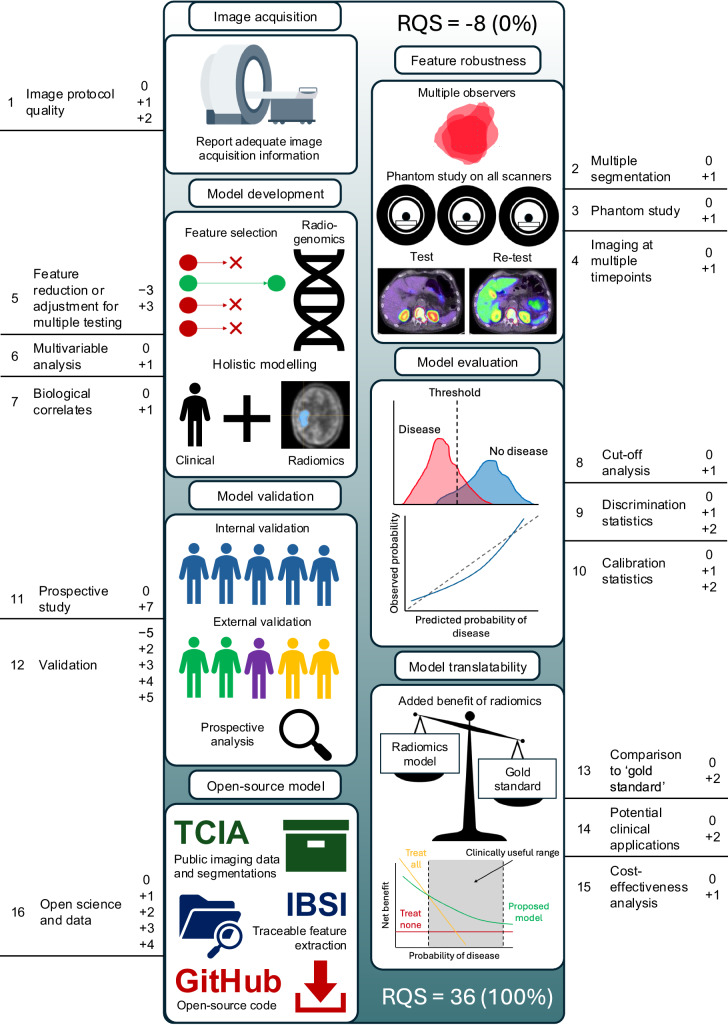
A visual guide to the Radiomics Quality Score (RQS). Beginning with a minimum score of −8 (0%), the 16 criteria of the RQS evaluate key points in the radiomics workflow to assess a model’s progress towards clinical translation. For each criterion, the potential attributable scores are shown, which sum to a maximum score of 36 (100%)

The Radiomics Quality Score (RQS) was an early attempt to guide researchers and reviewers in the appraisal of radiomics studies with respect to an ideal workflow [23]. The score, ranging from −8 to 36 (0% to 100%), encompasses 16 criteria, each one evaluating an aspect of reliably conducting and reporting a radiomics study, assigning/deducting varying point values (Fig. 1). The RQS addresses transparency in reporting image acquisition protocols, validation with multiple external datasets, development of prospective studies, correlation of imaging features with underlying biological mechanisms, appropriate evaluation metrics, and encourages open-source code and public datasets (Supplementary Table 1). Notably, external validation and prospective studies, which can be assigned five and seven points, respectively, are highly weighted.

Since its inception, the RQS has become the de facto appraisal tool for radiomics studies. A systematic review of the application of the RQS has been previously published by Spadarella et al [24], evaluating articles until the end of 2021. However, this study only reported the mean RQS from each included review and did not perform a meta-analysis of the RQS when applied to individual radiomics studies, which would provide a deeper understanding of adherence to this tool and its criteria. Additionally, many systematic reviews have been published since the end of 2021. To investigate if researchers are publishing radiomics studies with higher quality scores over time, the present work provides an updated systematic review and meta-analysis of the RQS. Here, we summarise how reviews approach study appraisal, any checklists (in addition to the RQS) used, reviewer criticisms of the RQS, average RQS and criteria scores, correlation of quality scores with study attributes (publication year, imaging modality, country), and inter-reader variability [25, 26]. As new appraisal tools emerge, such as the METhodological RadiomICs Score (METRICS) [27], this analysis may serve as a benchmark to evaluate their adoption within the radiomics community.

## Methods and materials

### Literature search

This systematic review and meta-analysis were registered (CRD42024493843) and conducted in accordance with the preferred reporting items for systematic reviews and meta-analyses (PRISMA) guidelines [28, 29]. Four databases were explored (Embase, Scopus, PubMed, Web of Science) using the keyword search: ((“radiomics” OR “radiomic”) AND “quality” AND “score”). We included systematic reviews published between January 1, 2022, and December 31, 2023, which implemented the RQS for quality assessment of radiomics studies. Additionally, we searched through Spadarella et al [24] for systematic reviews published before 2022. Excluded from analysis were reviews that modified the RQS or radiomics studies that were assigned “n/a” for any of the 16 criteria. Reviews with less than five papers, duplicates, editorials, letters, non-systematic literature reviews, and non-English articles were also excluded. Abstract screening and full-text review were conducted by two authors (N.B. and J.K.), and any disagreements were resolved through discussion.

### Data extraction

Here we define “quality assessment” as a single application of the RQS by an author of a review (herein referred to as a “reader”) resulting in a score out of 36 assigned to a radiomics study. For each review, the following was extracted (N.B.): first author, publication year, journal of publication, journal impact factor [30], disease (oncology/non-oncology), system/body location and organ investigated (assigned “multiorgan” if spanning numerous sites/diseases), number of quality assessments, number of readers, method to finalise the RQS from multiple readers, and other checklists used e.g., for assessing bias. Criticisms of the RQS in the Discussion section were noted and categorised. Additionally, there are circumstances where a radiomics paper may receive multiple quality assessments; either when the radiomics study develops multiple models and readers assign an RQS to each, or readers from separate systematic reviews assign an RQS to the same radiomics study. For simplicity, we treat these as independent scores.

For the meta-analysis, we attempted to extract increasingly granular levels of data from each included review: (1) descriptive statistics of overall mean or median RQS, (2) the RQS and the 16 criteria scores assigned to individual radiomics papers (quality assessments), and (3) quality assessments from multiple readers (each reader must rate all studies). When quality assessments are provided for multiple readers, the meta-analysis for the criteria scores could be confounded if a consensus was not used to finalise the scores (e.g., averaging criteria scores). Therefore, this process was standardised by using the consensus if provided, or else the most experienced reader’s scores; if experience was not specified, the first reader was used. For each quality assessment, we extracted the publication year of the respective radiomics study, to correlate quality assessments with time. Note this publication year is *not* from the review it’s extracted from. Similarly, where available for reviewed studies, we extracted information on the imaging modalities investigated and the country in which the study was conducted.

### Correction of assigned criteria scores

Extracted criteria scores underwent an integrity check. Firstly, we ensured that readers provided valid scores for each RQS criteria, e.g., criterion five (feature reduction) can be awarded −3 or +3; however, if a 0 is awarded, this is corrected to −3. Secondly, we summated the criteria, even if no correction was applied, to also confirm that this matched with the reported RQS. To ensure consistent application of the RQS across reviews, we only used the correctly scored and summated criteria in the meta-analysis. Additionally, the calculated mean RQS was compared to the originally reported mean RQS in reviews that provided criteria data, and the absolute difference was correlated with the journal impact factor.

### Statistical analysis

Descriptive statistics are presented as mean ± standard deviation (SD) (95% confidence interval (CI)) and median (range). Note that the meta-analysis pools together quality assessments from individual radiomics studies to calculate the mean RQS (mean ± SD (95% CI)). The average score for each criterion was calculated and reported as a fraction of the maximum achievable score and adherence (a binary measure when a criterion is assigned a score > 0). Although not reported here, a mean RQS was assigned to each included review, as illustrated in Supplementary Fig. 1, and in rare circumstances, the assigned mean RQS was estimated from the reported median RQS [31].

In the sub-group analysis, quality assessments of individual radiomics studies inherited the attributes of the review they were extracted from, which are: (1) if the review was originally included by Spadarella et al [24], (2) if the review was on oncology cohorts, and (3) the system/body location the review was categorised into. Additionally, the mean RQS of radiomics studies before and after the original RQS publication [23] was compared. For simplicity, since the RQS was published in October 2017, we treat radiomics studies published from 2018 onwards as published after the RQS. Again, the year of publication was *not* inherited from the review a quality assessment was extracted from. In the analysis of imaging modalities, quality assessments were grouped by the modality investigated. For multi-modality investigations, the quality assessment is evaluated for each modality investigated, e.g., a quality assessment from a positron emission tomography (PET)/magnetic resonance imaging (MRI) study would be included in the statistics for both PET and MRI.

Assessing inter-reader agreement of the criteria was by the quadratically weighted Cohen’s kappa statistic, averaged across pairwise combinations of reader scores [32]. A two-way random (absolute agreement, single rater/measurement) intraclass correlation coefficient (ICC) was calculated to assess reader agreement with the overall RQS [33, 34]. Classification of agreement is based on recommendations by Koo and Li, which was conservatively assessed using the lower 95% CI [35].

Correlations were measured using Pearson correlation. The Wilcoxon Rank Sum test was used for comparing sub-grouped quality assessments. The Kruskal-Wallis test was used when comparing multiple groups. Differences between calculated and reported mean RQS were assessed using Bland-Altman analysis. A *p* < 0.05 was considered significant for all statistical tests. Bonferroni correction was applied to adjust for multiple testing where necessary. Further information about what data is used for each analysis is illustrated in Supplementary Fig. 2.

## Results

### Literature search

**Fig. 2:**
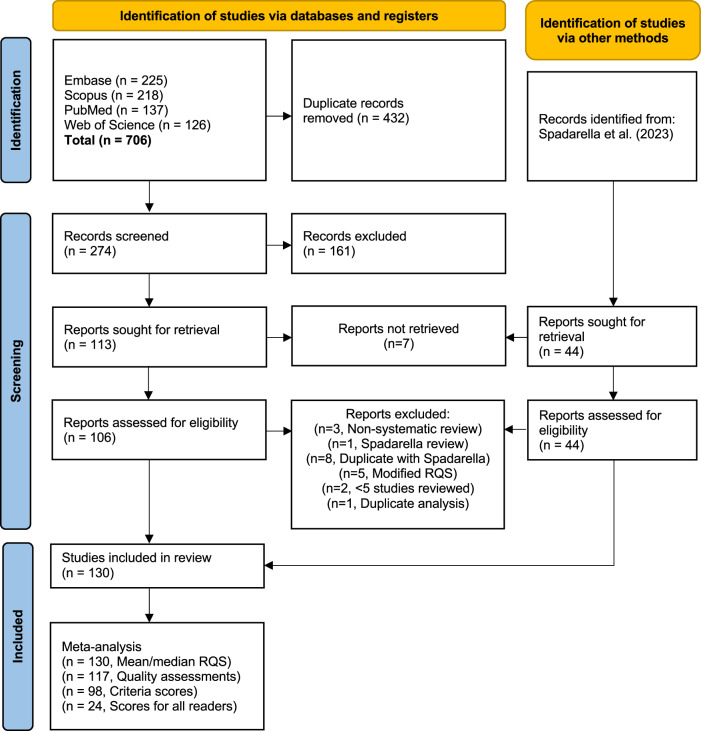
PRISMA diagram of literature search, abstract screening, and full-text review. Papers prior to 2022 were retrieved from Spadarella et al [24]. Further breakdown of the level of data provided in the included reviews, used in the meta-analysis, is shown

**Fig. 3:**
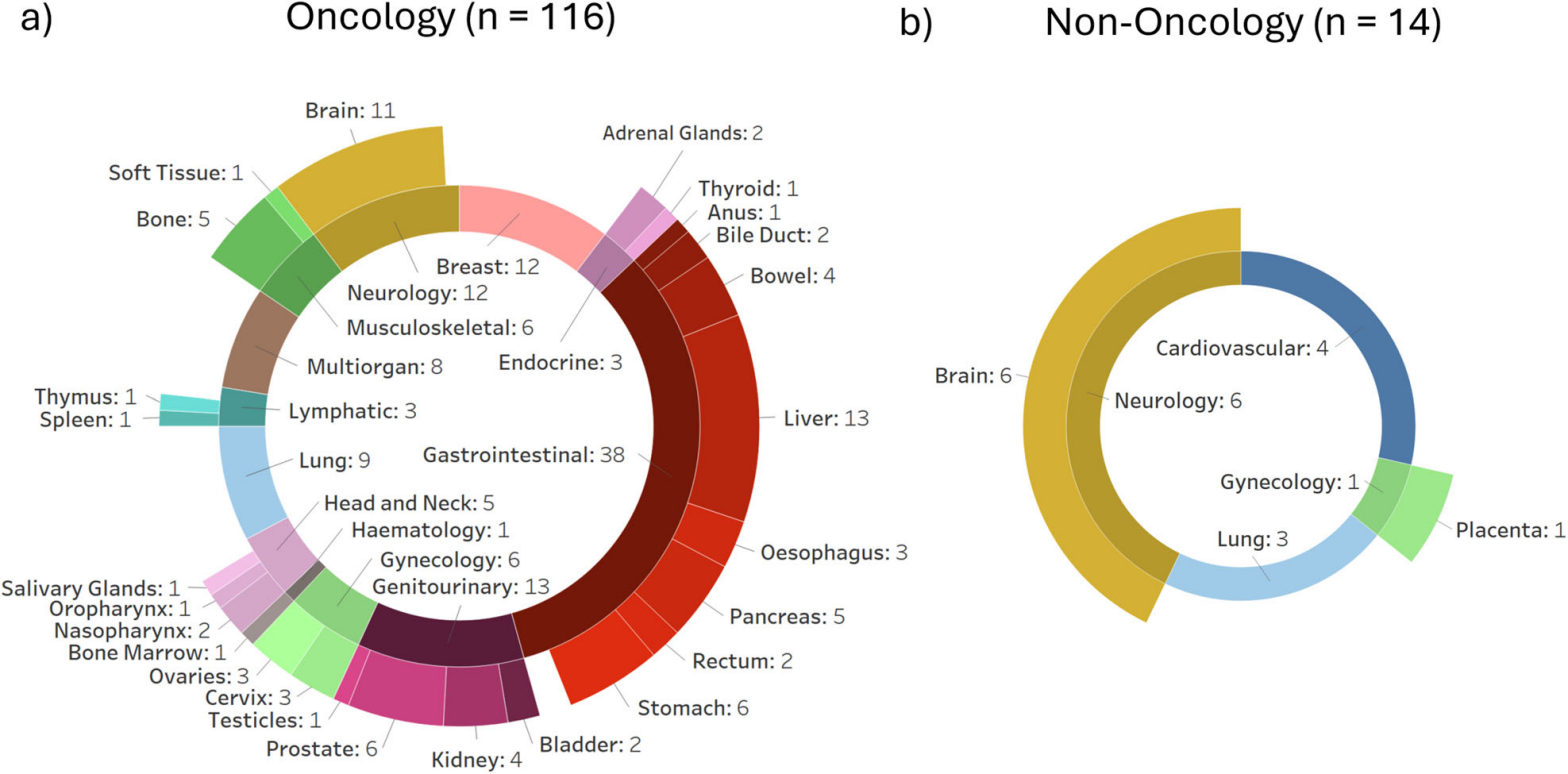
A total of 130 systematic reviews are shown, based on (**a**) those that included oncology studies and (**b**) those that did not. Categorisation of included reviews is by system/body location (inner circle), further split up by organ (outer circle)

The resulting article search is shown in the PRISMA diagram in Fig. 2. In this review, we included 89 articles published between January 1, 2022, and December 31, 2023. Additionally, 41 articles prior to 2022 were included from Spadarella et al [24], which brought the total to 130 systematic reviews (Supplementary Table 2). One journal was too new to extract a reliable impact factor. The topics covered by each review are summarised in Fig. 3, with gastrointestinal cancer (38/130, 29.2%) found to be the most reviewed application of radiomics.

### Data extraction

The median number of quality assessments conducted for each review was 22.5 (range: 6–190). A mean RQS was extracted from all 130/130 (100%) systematic reviews, with the mean estimated from the reported median in 4/130 (3.1%) (Supplementary Table 2). Individual quality assessments were provided in 117/130 (90.0%) reviews, where a total of 3258 quality assessments were extracted, with the associated year of publication. A subset of these reviews also included quality assessments with criteria scores (98/130, 75.4%) and scores from multiple readers (24/130, 18.5%). A total of 116 (89.2%) review papers included oncology cohorts in their articles. A summary of how reviews approach the quality assessment of articles can be found in Table 1. Reviews commonly used two readers when applying the RQS (108/130, 83.1%), with the final score decided by consensus (92/130, 70.8%), often arbitrated by a third reader.

**Table 1:** Review approaches to quality assessment

Review attribute	*n* (%)
No. of readers	
Two	108 (83.1%)
Three	7 (5.4%)
Four	4 (3.1%)
Five	1 (0.8%)
Not specified	10 (7.7%)
Method to finalise RQS	
Consensus	92 (70.8%)
Average	12 (9.2%)
Most experience	7 (5.4%)
Separate	2 (1.5%)
Not specified	17 (13.1%)
Additional checklist(s) used	
None	55 (42.3%)
QUADAS-2	51 (39.2%)
TRIPOD	21 (16.2%)
PROBAST	12 (9.2%)
IBSI	9 (6.9%)
CLAIM	4 (3.1%)
QUIPS	2 (1.5%)
MINIMAR	1 (0.8%)
NOS	1 (0.8%)

The number of readers used for quality assessment, method to finalise quality scores, and checklists used in conjunction with the Radiomics Quality Score are summarised

*RQS* Radiomics Quality Score, *QUADAS-2* quality assessment of diagnostic accuracy studies version 2 [36], *TRIPOD* transparent reporting of a multivariable prediction model for individual prognosis or diagnosis [37], *PROBAST* prediction model risk of bias assessment tool [38], *IBSI* image biomarker standardisation initiative, *CLAIM* CheckList for Artificial Intelligence in Medical imaging [48], *QUIPS* quality in prognosis studies [49], *MINIMAR* minimum information for medical artificial intelligence reporting [50], *NOS* Newcastle-Ottawa scale [51]

For other checklists used in conjunction with the RQS, the most common were QUADAS-2 (51/130, 39.2%) [36], TRIPOD (21/130, 16.2%) [37], and PROBAST (12/130, 9.2%) [38], with 55/130 (42.3%) using the RQS alone (see Table 1 for checklist definitions). Criticisms of the RQS were identified in 60/130 (46.2%) reviews and are summarised in Table 2. Criticisms on one or more criteria of the RQS in these reviews were broadly categorised as difficult to implement (21/60, 35.0%), not applicable (17/60, 28.3%), not reproducible (16/60, 26.7%), and too penalising (10/60, 16.7%). Additionally, there were overall criticisms that the RQS requires revisions (18/60, 30.0%), is not endorsed by the scientific community (3/60, 5.0%), and was not validated when initially published (1/60, 1.7%).

**Table 2:** Summary of review comments on the limitations of the Radiomics Quality Score (RQS)

Category	Description	*n* (%)
Difficult to implement	One or more of the criteria are too unrealistic or idealistic to be implemented in practice.	21 (35.0%)
Revisions required	The RQS needs to be revised or to have incremental updates.	18 (30.0%)
Applicability	One or more of the criteria cannot be applied under certain study contexts.	17 (28.3%)
Reproducibility	One or more of the criteria definitions are unclear, difficult to interpret, or vary greatly between readers.	16 (26.7%)
Too penalising	The weightings of one or more of the criteria are too harsh for development studies.	10 (16.7%)
Not endorsed by scientific community	The RQS has not been adopted or endorsed by the wider scientific community.	3 (5.0%)
Not validated	The RQS was not validated when initially published.	1 (1.7%)

Criticisms of the RQS within the Discussion section of 60 included reviews were noted and categorised. No criticisms were found in the remaining reviews

### Correction of assigned criteria scores

The existence of errors when criteria scores were (potentially) corrected and summated was found in 39/98 (39.8%) reviews. The extent of these errors, via Bland-Altman analysis, revealed a slight positive bias, i.e., overestimation (mean, 0.3; limits of agreement, −1.7 to 2.4) when applying the RQS (Supplementary Fig. 3). These errors were not found to be correlated with journal impact factor (Pearson *R* = −0.1, *p* = 0.76; Supplementary Fig. 4).

### Meta-analysis

When grouping all quality assessments from 117 reviews, the overall mean RQS was 9.4 ± 6.4 (9.1–9.6) (26.1% ±17.8% (25.3%–26.7%)). Subgroup comparison of the mean RQS is shown in Table 3. The mean RQS was significantly greater in recent reviews included in our literature search (starting from 2022) compared to the reviews originally included by Spadarella et al [24] (7.1 ± 6.2 (6.8–7.5) vs. 10.6 ± 6.1 (10.3–10.8), *p* < 0.001). No significant difference between oncology and non-oncology reviews (oncology, 9.4 ± 6.4 (9.2–9.6); non-oncology, 8.9 ± 5.6 (8.1–9.8); *p* > 0.99) was observed.

**Table 3:** Radiomics Quality Score subgroup comparison

Subgroup (*n*)		*p*-value^a^
Spadarella et al [24] (1121)	7.1 ± 6.2 (6.8–7.5)	< 0.001
Oncology (3076)	9.4 ± 6.4 (9.2–9.6)	> 0.99
System/body location^b^		
Breast (231)	13.1 ± 5.8 (12.4–13.9)	< 0.001
Cardiovascular (72)	7.5 ± 6.1 (6.0–8.9)	0.13
Endocrine (78)	7.6 ± 8.1 (5.8–9.4)	> 0.99
Gastrointestinal (1014)	9.6 ± 6.5 (9.2–10.0)	0.25
Genitourinary (452)	8.8 ± 6.1 (8.2–9.4)	> 0.99
Gynaecology (213)	8.7 ± 6.5 (7.8–9.6)	> 0.99
Head and Neck (86)	10.3 ± 5.8 (9.0–11.5)	> 0.99
Haematology (23)	10.0 ± 3.9 (8.4–11.7)	> 0.99
Lung (299)	9.1 ± 6.5 (8.3–9.8)	> 0.99
Lymphatic (73)	7.0 ± 6.1 (5.6–8.5)	0.04
Musculoskeletal (75)	9.3 ± 5.3 (8.0–10.5)	> 0.99
Neurology (404)	8.0 ± 5.8 (7.4–8.5)	< 0.001

Data is shown as mean ± standard deviation (95% confidence interval)

^a^ Rank Sum test against quality assessments not in subgroup, adjusted for multiple testing

^b^ Rank Sum test excludes multiorgan reviews

Subgroup comparison by reviewed system/body location found the mean RQS of breast imaging to be significantly higher than other areas of review (breast, 13.1 ± 5.8 (12.4–13.9); non-breast, 8.9 ± 6.3 (8.7–9.2); *p* < 0.001). Lymphatic (lymphatic, 7.0 ± 6.1 (5.6–8.5); non-lymphatic, 9.3 ± 6.4 (9.1–9.6); *p* = 0.04) and neuro-imaging (neurology, 8.0 ± 5.8 (7.4–8.5); non-neurology, 9.5 ± 6.5 (9.2–9.7); *p* < 0.001) were significantly lower. These comparisons excluded multiorgan reviews. When restricting the neuro-imaging comparison to only the reviews included by Spadarella et al [24], significance was not reached (neurology, 6.7 ± 5.2 (5.1–8.3); non-neurology, 7.2 ± 6.3 (6.8–7.6); *p* = 0.56). The distribution of quality assessments and average criteria scores sub-grouped by system/body location can be found in Supplementary Fig. 5 and Supplementary Fig. 6, respectively.

**Fig. 4:**
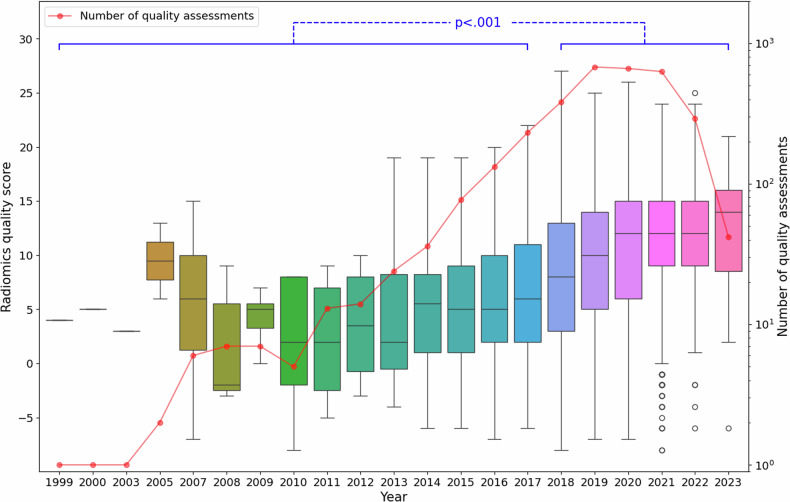
Trend of the Radiomics Quality Score over time. Boxplots illustrate the distribution of individual quality assessments grouped by the year of publication of the respective radiomics study. The number of quality assessments conducted each year is illustrated by the line plot. A significant positive correlation between quality scores with time is evident (Pearson *R* = 0.32, *p* < 0.001) and is significantly higher (*p* < 0.001) after the publication of the original paper by Lambin et al in 2017 [23]

The publication year of radiomics studies was obtained for every quality assessment and ranged from 1999 to 2023. Imaging modality and country of origin were extracted for 2981 (91.5%) and 861 (26.4%) quality assessments, respectively. The distribution of quality assessments over time is shown in Fig. 4 and was found to be positively correlated with time (Pearson *R* = 0.32, *p* < 0.001) and significantly higher after the publication of the original paper by Lambin et al [23] (year < 2018, 5.6 ± 6.1 (5.1–6.1); year ≥ 2018, 10.1 ± 6.1 (9.9–10.4); *p* < 0.001). A total of 233/3258 (7.2%) scores were ≥ 50% of the maximum RQS (Supplementary Fig. 7). Quality assessments of radiomics studies that investigated computed tomography (CT), MRI, PET, and ultrasound (US) were found to be significantly different (*p* < 0.001), with US studies generally exhibiting the highest mean RQS (11.4), followed by MRI (9.7) and CT (9.3), with the lowest from PET studies (7.4), as shown in Table 4. Although mammography studies exhibited the highest mean RQS out of all modalities (16.1), the sample size was limited (*n* = 14). The distribution of these quality assessments by imaging modality can be found in Supplementary Fig. 8. Distribution of quality assessments by country can be found in Supplementary Fig. 9.

**Table 4:** A summary of quality assessments by imaging modality investigated

Modality	*n* (%)	Mean ± SD
CT	1557 (52.20%)	9.26 ± 6.44
MRI	1287 (43.17%)	9.73 ± 6.19
PET	266 (8.92%)	7.41 ± 7.06
US	79 (2.65%)	11.41 ± 6.84
		*p* < 0.001^a^
Other		
MMG	14 (0.47%)	16.14 ± 6.37
SPECT	2 (0.07%)	10.50 ± 2.12
DBT	1 (0.03%)	21.00

These quality assessments are grouped by CT, MRI, PET, and US and may be included in more than one group, e.g., PET/MRI, PET/CT, and CT/MRI studies. The results for other imaging modalities with minimal sample numbers (MMG, SPECT, DBT) are also shown

*SD* standard deviation, *CT* computed tomography, *MRI* magnetic resonance imaging, *PET* positron emission tomography, *US* ultrasound, *MMG* mammography, *SPECT* single-photon emission computed tomography, *DBT* digital breast tomosynthesis

^a^ Kruskal–Wallis test

**Fig. 5:**
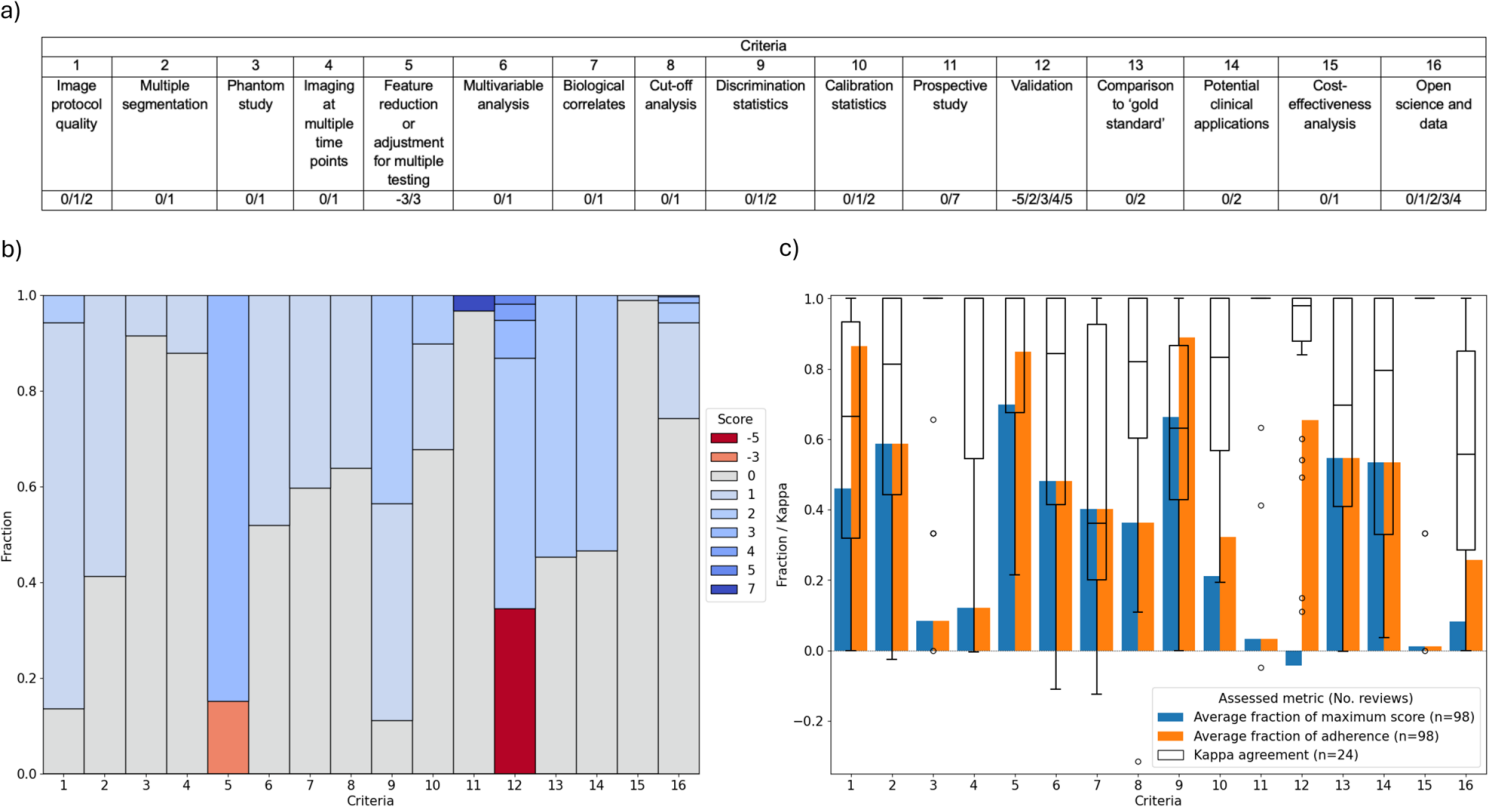
Criteria analysis of included reviews. In (**a**) criteria definitions and available scores are shown with (**b**) the overall distribution of these scores by reviews, and **c** the average fraction of the maximum score and adherence (assigned score > 0) is calculated from 97 reviews, and observer agreement spread from 24 reviews is overlayed with boxplots

**Fig. 6:**
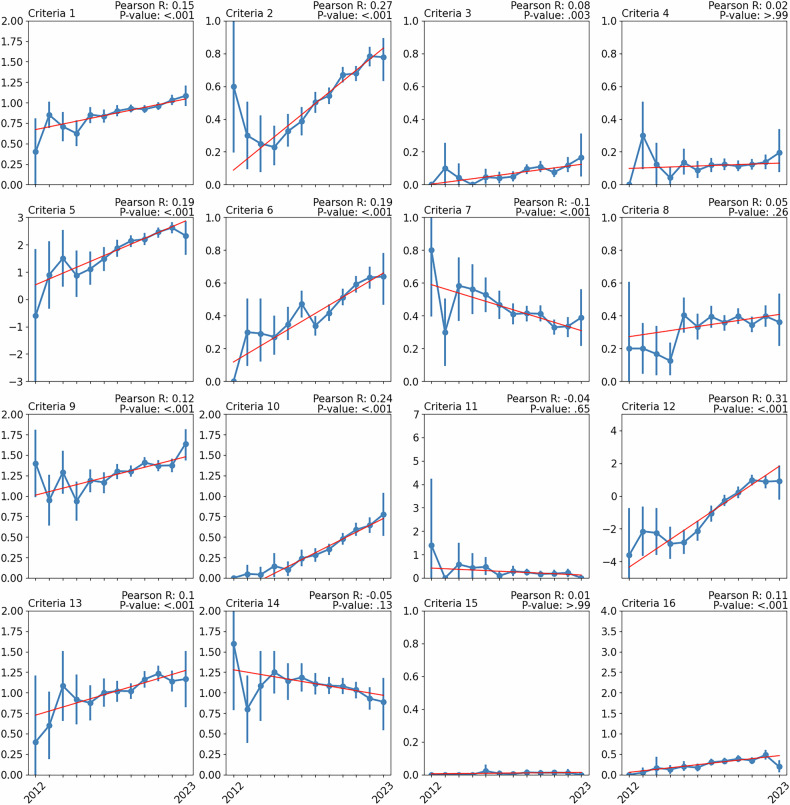
Change in criteria scores over time from 2012 to 2023. Scores for each year are represented as mean and 95% confidence intervals. A line of best fit is overlayed to illustrate the trend over time, with calculated Pearson correlation and associated *p*-value (corrected for multiple testing). Data prior to 2012 has been excluded due to insufficient sample sizes

Inter-reader agreement of the RQS from 24 reviews can be found in Table 5 (with further detail in Supplementary Fig. 10). Overall, 9/24 (37.5%) reviews exhibited “good” or “excellent” agreement. The average fraction of the maximum score and adherence for all criteria are shown in Fig. 5, including inter-reader analysis. The average fraction of the maximum score and adherence ranged from −4.2% to 69.9% and 1.1%–88.9%, respectively. Twenty-four kappa values were calculated for each criterion, with the median kappa across the 16 criteria ranging from 0.36–1.00. Additionally, the correlation of these criteria over time is shown in Fig. 6. Correlation analysis found 10/16 (62.5%) were positively correlated, 1/16 (6.3%) were negatively correlated, and 5/16 (31.3%) had no correlation with time, respectively, after adjustment for multiple testing.

**Table 5:** Observer agreement in applying the Radiomics Quality Score

Level of agreement^a^	Criteria^b^	*n* (%)
Excellent	ICC ≥ 0.90	6 (25.0%)
Good	0.75 ≤ ICC < 0.90	3 (12.5%)
Moderate	0.5 ≤ ICC < 0.75	6 (25.0%)
Poor	ICC < 0.50	9 (37.5%)

Agreement is calculated from reviews that supplied quality assessment data for multiple readers (*n* = 24)

*ICC* intraclass correlation coefficient

^a^ According to the lower bound of the 95% confidence interval

^b^ As recommended by Koo and Li [35]

## Discussion

This systematic review and meta-analysis provide a summary insight into the adherence of radiomics studies to the RQS. Criticism of the RQS was found in 60/130 (46.2%) of review papers, and more than half of review papers utilised other evaluation tools and checklists. Readers incorrectly applied or summated RQS criteria in almost 40% of the 98 reviews investigated. The mean RQS of quality assessments from 117 review papers was 9.4 (26.1%) and temporal analysis indicates the RQS of radiomics studies has increased with time, along with improvements in 10/16 criteria. Radiomics studies investigating US exhibited the highest mean RQS, followed by MRI, CT, and lastly PET.

Progress on implementing phantom studies, test-retest studies, external validation, prospective studies, cost-effectiveness analysis, and open science in radiomics studies is minimal or insignificant. Additionally, investigating biological correlates appears to be significantly decreasing over time. Researchers may consider these components of the RQS as difficult to implement, non-applicable, or not relevant to radiomics analysis. Phantom, test–retest, and prospective studies are resource-intensive, which is impractical when most radiomics papers are explorative and for development purposes. Furthermore, since most studies are developmental, cost-effectiveness analysis is non-applicable to most researchers, which would occur in the latter stages of clinical translation. Open science appears to not be prioritised by researchers; however, most studies utilise image biomarker standardisation initiative (IBSI) compliant radiomics extraction software [22]. Notably, external validation and prospective study criteria form one-third of the maximum score (12/36), which developmental studies won’t receive, echoing the criticism that some criteria are “too penalising”. Lastly, there is a potential trend in biological correlates being viewed as less relevant to radiomics analysis by researchers. Therefore, priorities in the radiomics community may be evolving.

Tomaszewski and Gillies [39] emphasised the importance of investigating the biological underpinnings of predictive radiomics features, and interest still remains in exploring the relationship between imaging characteristics and tumour genetic expression [40, 41]; however, recent community guidelines and initiatives do not appear to exhibit the same emphasis for clinical translation [27, 42–44]. In particular, the METRICS tool represents an important community initiative on radiomics study evaluation, which does not include biological correlates [27]. Where the points for the RQS criteria are arbitrary, the METRICS tool weighs items based on expert opinion. Interestingly, the category in METRICS with the lowest weight is open science, which reflects our observations, although its inclusion still reflects its importance to the radiomics community. Lastly, the applicability of the RQS to deep learning studies has also been addressed as a conditional item for the use of end-to-end deep learning pipelines, providing flexibility to authors who may wish to investigate hand-crafted radiomics features, automatically learned features by neural networks or both. Other resources also continue to evolve, for example, CheckList for Artificial Intelligence in Medical imaging (CLAIM) received an update in 2024 and phase two of the IBSI addressed standardisation of imaging filters [42, 45].

Naturally, our results can be compared to Spadarella et al, which included 44 systematic reviews in their analysis [24]. Like their study, we found no significant difference in mean RQS between oncology and non-oncology-focused reviews. In contrast, reviews of neuro-imaging applications of radiomics had a significantly lower mean RQS when compared to other imaging areas. This significance was not observed when repeating this analysis on data extracted from only reviews included by Spadarella et al [24], so we extended the subgroup analysis to each criterion for insight. Neurology was notably low for multivariable analysis and comparison to a “gold standard”; therefore, further focus may be required in these areas. Additionally, neuro-oncology does not have a clear “gold standard” to compare to, such as TNM staging, which may partially explain poor performance due to “applicability”. In comparison, breast imaging studies were significantly higher and were more likely to conduct reproducibility analyses, feature selection, biological correlation, comparison to a “gold standard”, and open science.

A significant difference in quality assessments was observed between radiomics studies which investigate different imaging modalities. Namely, we compared the most investigated modalities with sufficient sample sizes: US, PET, CT, and MRI. Notably, studies extracting features from US tended to exhibit a higher mean RQS, which may be because US is often readily available, non-ionising, and has short scan times. PET radiomics studies exhibited the lowest mean RQS, which, in contrast to US, is resource-intensive, ionising, and has long scan times. As such, these attributes may impact dataset sizes and the implementation of reproducibility studies, resulting in a lower RQS.

A reproducibility study of the RQS by D’Antonoli et al found poor-to-moderate ICC agreement of quality scores amongst observers, independent of their experience and initial training [26]. Kappa values of the criteria ranged from −0.21 to 0.75. The agreement, as measured by the ICC and kappa, appears lower compared to the agreement reported in the present analysis, potentially highlighting an inherent bias in self-reported agreement within institutions. These results reflect the “reproducibility” criticism due to purported unclear criteria definitions. Indeed, a lack of inter-reader agreement was noted in the earliest use of the RQS [25]. Since some measurements of the agreement were based on criteria that are rarely implemented in radiomics studies, we reported kappa values alongside observed adherence. As expected, agreement is generally high with these criteria. Furthermore, kappa values are unstable with skewed marginal distributions (prevalence) of ratings [46, 47].

There are some limitations to this meta-analysis. Firstly, we did not account for overlap in quality assessments of radiomics studies by readers in separate reviews. Secondly, we relied on reviews to accurately report the publication year, imaging modality, and country of radiomics studies. Thirdly, excluded systematic reviews that omit the RQS may include studies of lower quality, resulting in selection bias. Additionally, a keyword search of “radiomics” since 2022 retrieved over 9400 studies indexed in Scopus, a majority of which would not have been assessed in the reviews we included. Fourthly, we attempted to correct errors in criteria application in reviews; however, the intent of the original score cannot be known with certainty. Nevertheless, we believe a consistent application of each criterion across reviews was required for the meta-analysis; an approach which subsequently revealed frequent, incorrect application of the RQS criteria. Importantly, all systematic reviews and reported quality assessments will include noise, and it has been demonstrated that applying the RQS to the same radiomics study is highly variable [26]. To overcome this, we extracted an extremely large sample size of quality assessments to robustly identify trends in the radiomics literature. Lastly, to better ensure accurate reporting in the future, our group has developed an RQS calculator (https://uwa-medical-physics-research-group.github.io/RQS-calculator/).

We’ve demonstrated that radiomics studies are increasingly adhering to the criteria of the RQS. However, the observed progress of a majority of studies to date has not demonstrated a sufficiently high level of evidence for clinical translation. The RQS has demonstrable shortcomings, and radiomics has rapidly evolved since its inception, spurring the emergence of new appraisal tools and community advancement. Importantly, if the field of radiomics can identify a small subset of features that are generalisable, robust, and predictive, which are then rigorously validated, clinical translation will be achievable.

## Supplementary information

ELECTRONIC SUPPLEMENTARY MATERIAL

## Abbreviations

CLAIM: CheckList for Artificial Intelligence in Medical imaging
IBSI: Image biomarker standardisation initiative
ICC: Intraclass correlation coefficient
METRICS: METhodological RadiomICs Score
PRISMA: Preferred reporting items for systematic reviews and meta-analyses
RQS: Radiomics Quality Score
