# Correction: Evaluating the impact of the radiomics quality score: a systematic review and meta-analysis

**DOI:** 10.1007/s00330-025-11570-9

**Published:** 2025-04-15

**Authors:** Nathaniel Barry, Jake Kendrick, Kaylee Molin, Suning Li, Pejman Rowshanfarzad, Ghulam M. Hassan, Jason Dowling, Paul M. Parizel, Michael S. Hofman, Martin A. Ebert

**Affiliations:** 1 School of Physics, Mathematics and Computing, University of Western Australia, Crawley, WA Australia; 2 Centre for Advanced Technologies in Cancer Research (CATCR), Perth, WA Australia; 3 Australian Centre for Quantitative Imaging, Medical School, University of Western Australia, Crawley, WA Australia; 4 Department of Radiation Oncology, Sir Charles Gairdner Hospital, Nedlands, WA Australia; 5 The Australian e-Health Research Centre, CSIRO, Brisbane, QLD Australia; 6 David Hartley Chair of Radiology, Royal Perth Hospital and University of Western Australia, Perth, WA Australia; 7 Medical School, University of Western Australia, Perth, WA Australia; 8 Prostate Cancer Theranostics and Imaging Centre of Excellence (ProsTIC), Molecular Imaging and Therapeutic Nuclear Medicine, Cancer Imaging, Peter MacCallum Centre, Melbourne, VIC Australia; 9 Sir Peter MacCallum Department of Oncology, University of Melbourne, Melbourne, VIC Australia

**Correction to: European Radiology**

10.1007/s00330-024-11341-y, published online 10 January 2025

In the original article, the submitted file for Fig. 6 depicted data which was outdated at the time of publication. The written results described in the original article remain correct; this updated Fig. 6 version now makes the article text consistent with the figure's content. Fig. 6 has been replaced with this updated version. The corresponding author apologises for this error.

The outdated Fig. 6 can be seen below:
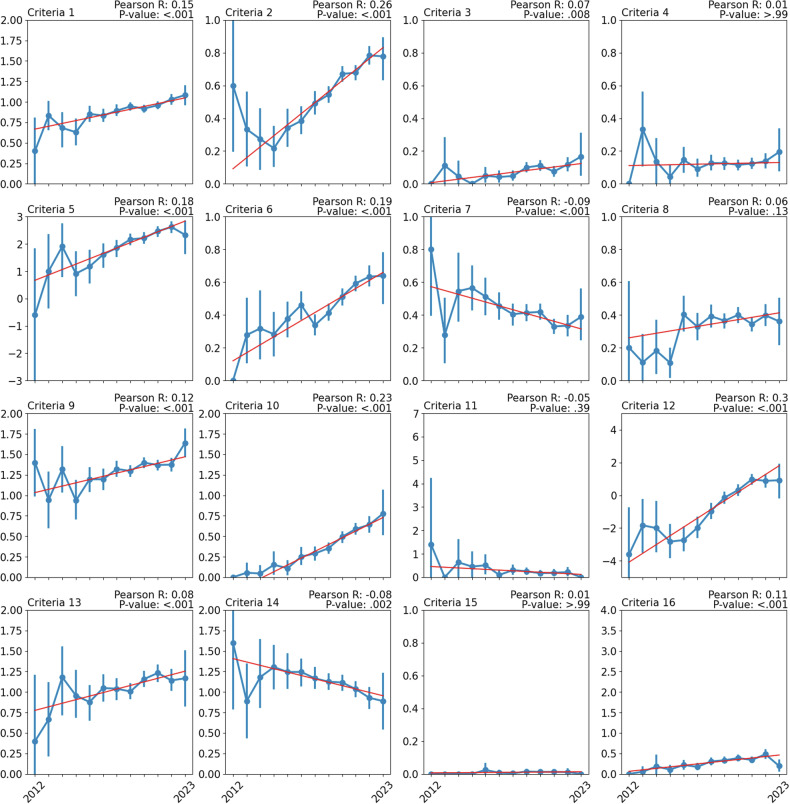


**Fig. 6** Change in criteria scores over time from 2012 to 2023. Scores for each year are represented as mean and 95% confidence intervals. A line of best fit is overlayed to illustrate the trend over time, with calculated Pearson correlation and associated *p*-value (corrected for multiple testing). Data prior to 2012 has been excluded due to insufficient sample sizes.

The updated Fig. 6 is as follows:
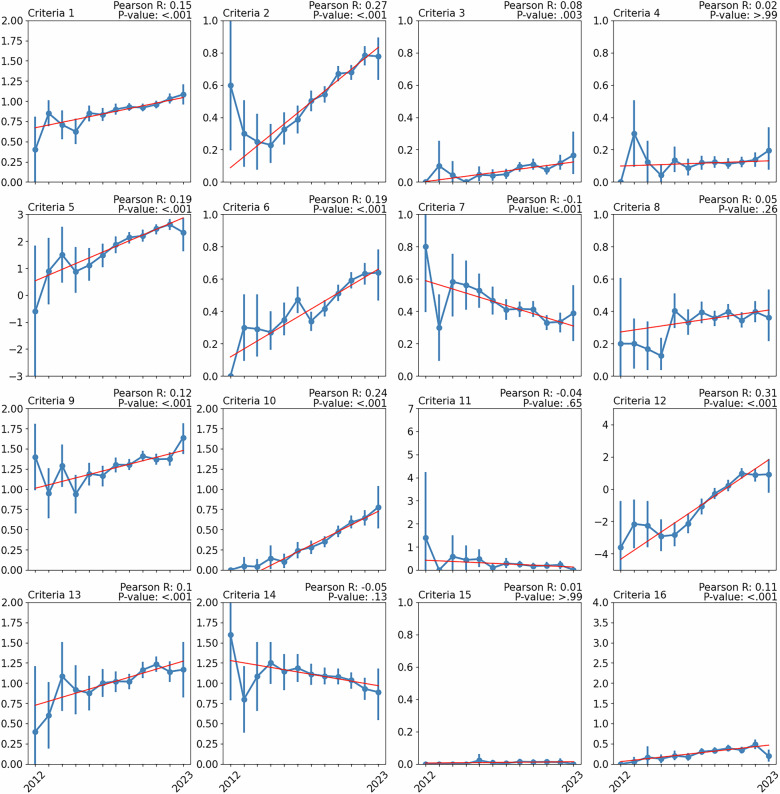


**Fig. 6** Change in criteria scores over time from 2012 to 2023. Scores for each year are represented as mean and 95% confidence intervals. A line of best fit is overlayed to illustrate the trend over time, with calculated Pearson correlation and associated *p*-value (corrected for multiple testing). Data prior to 2012 has been excluded due to insufficient sample sizes.

The original article has been corrected.

