# Supplementary material for: Evaluating the impact of the Radiomics Quality Score: a systematic review and meta-analysis
Source: Eur Radiol. 2025 Jan 10;35(3):1701–13. doi: 10.1007/s00330-024-11341-y (PMC11835903; doi:10.1007/s00330-024-11341-y)
Supplement: Supplementary file 1 — ELECTRONIC SUPPLEMENTARY MATERIAL [file 330_2024_11341_MOESM1_ESM.docx]

**Supplementary Table 1** Radiomics quality score criteria.

| **Criteria** | | **Points** |
| --- | --- | --- |
| **1** | Image protocol quality - well-documented image protocols (for example, contrast, slice thickness, energy, etc.) and/or usage of public image protocols allow reproducibility/replicability | + 1 (if protocols are well-documented)  + 1 (if public protocol is used) |
| **2** | Multiple segmentations - possible actions are: segmentation by different physicians/algorithms/software, perturbing segmentations by (random) noise, segmentation at different breathing cycles. Analyse feature robustness to segmentation variabilities | + 1 |
| **3** | Phantom study on all scanners - detect inter-scanner differences and vendor-dependent features. Analyse feature robustness to these sources of variability | + 1 |
| **4** | Imaging at multiple time points - collect images of individuals at additional time points. Analyse feature robustness to temporal variabilities (for example, organ movement, organ expansion/shrinkage) | + 1 |
| **5** | Feature reduction or adjustment for multiple testing - decreases the risk of overfitting. Overfitting is inevitable if the number of features exceeds the number of samples. Consider feature robustness when selecting features | − 3 (if neither measure is implemented)  + 3 (if either measure is implemented) |
| **6** | Multivariable analysis with non radiomics features (for example, EGFR mutation) - is expected to provide a more holistic model. Permits correlating/inferencing between radiomics and non radiomics features | + 1 |
| **7** | Detect and discuss biological correlates - demonstration of phenotypic differences (possibly associated with underlying gene–protein expression patterns) deepens understanding of radiomics and biology | + 1 |
| **8** | Cut-off analyses - determine risk groups by either the median, a previously published cut-off or report a continuous risk variable. Reduces the risk of reporting overly optimistic results | + 1 |
| **9** | Discrimination statistics - report discrimination statistics (for example, C-statistic, ROC curve, AUC) and their statistical significance (for example, p-values, confidence intervals). One can also apply resampling method (for example, bootstrapping, cross-validation) | + 1 (if a discrimination statistic and its statistical significance are reported)  + 1 (if a resampling method technique is also applied) |
| **10** | Calibration statistics - report calibration statistics (for example, Calibration-in-the-large/slope, calibration plots) and their statistical significance (for example, *P*-values, confidence intervals). One can also apply resampling method (for example, bootstrapping, cross-validation) | + 1 (if a calibration statistic and its statistical significance are reported)  + 1 (if a resampling method technique is also applied) |
| **11** | Prospective study registered in a trial database - provides the highest level of evidence supporting the clinical validity and usefulness of the radiomics biomarker | + 7 (for prospective validation of a radiomics signature in an appropriate trial) |
| **12** | Validation - the validation is performed without retraining and without adaptation of the cut-off value, provides crucial information with regard to credible clinical performance | - 5 (if validation is missing)  + 2 (if validation is based on a dataset from the same institute)  + 3 (if validation is based on a dataset from another institute)  + 4 (if validation is based on two datasets from two distinct institutes)  + 4 (if the study validates a previously published signature)  + 5 (if validation is based on three or more datasets from distinct institutes)  *Datasets should be of comparable size and should have at least 10 events per model feature |
| **13** | Comparison to 'gold standard' - assess the extent to which the model agrees with/is superior to the current 'gold standard' method (for example, TNM-staging for survival prediction). This comparison shows the added value of radiomics | + 2 |
| **14** | Potential clinical utility - report on the current and potential application of the model in a clinical setting (for example, decision curve analysis). | + 2 |
| **15** | Cost-effectiveness analysis - report on the cost-effectiveness of the clinical application (for example, QALYs generated) | + 1 |
| **16** | Open science and data - make code and data publicly available. Open science facilitates knowledge transfer and reproducibility of the study | + 1 (if scans are open source)  + 1 (if region of interest segmentations are open source)  + 1 (if code is open source)  + 1 (if radiomics features are calculated on a set of representative ROIs and the calculated features and representative ROIs are open source) |
|  | Total points (36 = 100%) | |

**Supplementary Fig. 1** Flowchart illustrating extraction of study characteristics and meta-analysis data from each included review. For each review, a mean RQS was assigned and, depending on the level of data provided by each review, the methodology differed. In brief, we prioritised calculating the mean RQS from raw and corrected data, rather than what was reported by each review. If only the median RQS was reported, the mean was estimated as in [1].

**Supplementary Fig. 2** Flowchart illustrating what data was used when undertaking each of the key analysis detailed in the main manuscript.

**Supplementary Table 2** Characteristics of included review papers.

| **First Author** | **Year** | **DOI** | **Journal** | **Impact Factor (JCR)** | **System/Body location** | **Organ** | **Oncology** | **No. quality assessments** | **No. readers*** | **Multireader data**** | **Final scores***** | **Criteria scores** | **Final score decider** | **Mean RQS** | **Other Checklists** |
| --- | --- | --- | --- | --- | --- | --- | --- | --- | --- | --- | --- | --- | --- | --- | --- |
| Abbas [2] | 2023 | 10.1016/j.ejro.2023.100511 | European Journal of Radiology Open | 2 | Multiorgan |  | Yes | 13 | 2 | No | Yes | Yes | Consensus | 11.08 |  |
| Abdurixiti [3] | 2021 | 10.1259/bjr.20201272 | The British Journal of Radiology | 2.8 | Lung |  | Yes | 6 | 2 | Yes | Yes | Yes | Average | 11 |  |
| Abunahel [4] | 2021 | 10.1007/s00330-020-07376-6 | European Radiology | 5.9 | Gastrointestinal | Pancreas | Yes | 72 | Not specified | No | Yes | Yes | Not specified | 7.46 |  |
| Adusumilli [5] | 2023 | 10.1186/s13244-023-01500-y | Insights into Imaging | 4.7 | Gynaecology | Ovaries | Yes | 33 | 2 | No | Yes | Yes | Consensus | 9.52 | QUIPS |
| Albalkhi [6] | 2023 | 10.1007/s00247-023-05679-6 | Pediatric Radiology | 2.3 | Neurology |  | Yes | 17 | 2 | No | Yes | Yes | Consensus | 12.12 | QUADAS-2 |
| Aringhieri [7] | 2022 | 10.3390/diagnostics12123002 | Diagnostics | 3.6 | Head and Neck | Salivary Glands | Yes | 23 | 2 | No | Yes | Yes | Consensus | 11.26 |  |
| Azadikhah [8] | 2022 | 10.1259/bjr.20211211 | British Journal of Radiology | 2.8 | Genitourinary | Kidney | Yes | 87 | 2 | Yes | Yes | Yes | Average | 14.24 |  |
| Bhandari [9] | 2021a | 10.1007/s00261-020-02832-9 | Abdominal Radiology | 2.4 | Genitourinary | Kidney | Yes | 13 | Not specified | No | Yes | No | Not specified | 11.31 |  |
| Bhandari [10] | 2021b | 10.3174/ajnr.A6875 | American Journal of Neuroradiology | 3.5 | Neurology | Brain | Yes | 14 | 2 | No | Yes | Yes | Consensus | 9.21 | QUADAS-2 |
| Bian [11] | 2023 | 10.3389/fnagi.2023.1199826 | Frontiers in Aging Neuroscience | 4.8 | Neurology | Brain | No | 28 | 2 | No | Yes | Yes | Consensus | 10.64 |  |
| Boca [12] | 2023 | 10.3390/diagnostics13132300 | Diagnostics | 3.6 | Genitourinary | Bladder | Yes | 26 | 2 | No | Yes | Yes | Consensus | 11.62 | QUADAS-2 |
| Brancato [13] | 2022 | 10.3390/cancers14112731 | Cancers | 5.2 | Neurology | Brain | Yes | 42 | 2 | No | Yes | Yes | Consensus | 8.26 |  |
| Calabrese [14] | 2021 | 10.1007/s00432-021-03606-6 | Journal of Cancer Research and Clinical Oncology | 3.6 | Breast |  | Yes | 10 | 2 | No | Yes | No | Not specified | 11.1 |  |
| Calimano-Ramirez [15] | 2023 | 10.1007/s00261-023-03924-y | Abdominal Radiology | 2.4 | Genitourinary | Prostate | Yes | 11 | 2 | Yes | Yes | Yes | Most experience | 15.91 |  |
| Cannella [16] | 2023 | 10.1186/s13244-023-01365-1 | Insights into Imaging | 4.7 | Gastrointestinal | Bile Duct | Yes | 38 | 3 | Yes | Yes | Yes | Separate | 7.67 |  |
| Carbonara [17] | 2021 | 10.1155/2021/5566508 | Journal of Oncology | 4.501 | Head and Neck |  | Yes | 8 | 4 | Yes | Yes | Yes | Average | 7.25 |  |
| Chang [18] | 2022 | 10.1007/s00330-022-08587-9 | European Radiology | 5.9 | Cardiovascular |  | No | 32 | 2 | No | No | No | Consensus | 5.16 | TRIPOD, IBSI |
| Chen [19] | 2021 | 10.1007/s00259-021-05509-7 | European Journal of Nuclear Medicine and Molecular Imaging | 9.1 | Lung |  | Yes | 15 | 2 | Yes | Yes | Yes | Average | 10.47 |  |
| Chen [20] | 2022 | 10.1007/s00330-022-08704-8 | European Radiology | 5.9 | Gastrointestinal | Stomach | Yes | 25 | 2 | No | Yes | Yes | Consensus | 14.16 |  |
| Chetan [21] | 2021 | 10.1007/s00330-020-07141-9 | European Radiology | 5.9 | Lung |  | Yes | 14 | Not specified | No | Yes | Yes | Not specified | 2.5 |  |
| Crombé [22] | 2020 | 10.1016/j.ejrad.2020.109283 | European Journal of Radiology | 3.3 | Musculoskeletal | Soft Tissue | Yes | 52 | 2 | No | No | No | Most experience | 4.75 | QUADAS-2, IBSI |
| Davey [23] | 2021 | 10.1016/j.ejrad.2021.109996 | European Journal of Radiology | 3.3 | Breast |  | Yes | 38 | 2 | No | Yes | No | Consensus | 17.58 |  |
| Dercle [24] | 2022 | 10.1136/jitc-2022-005292 | Journal for ImmunoTherapy of Cancer | 10.9 | Multiorgan |  | Yes | 79 | Not specified | No | Yes | No | Not specified | 13.51 |  |
| Dragoș [25] | 2023 | 10.3390/diagnostics13050857 | Diagnostics | 3.6 | Neurology | Brain | No | 6 | 2 | No | Yes | Yes | Consensus | 10.83 | PROBAST |
| Du [26] | 2023 | 10.1007/s11604-022-01352-4 | Japanese Journal of Radiology | 2.1 | Gastrointestinal | Stomach | Yes | 27 | Not specified | No | Yes | No | Not specified | 13.41 |  |
| Faiella [27] | 2022 | 10.3390/ijerph19031880 | International Journal of Environmental Research and Public Health | 4.614 | Musculoskeletal | Bone | Yes | 13 | 2 | No | Yes | No | Consensus | 8.15 |  |
| Faiella [28] | 2023 | 10.3390/jcm12227032 | Journal of Clinical Medicine | 3.9 | Genitourinary | Prostate | Yes | 16 | 2 | No | Yes | No | Not specified | 13.94 |  |
| Fanni [29] | 2023a | 10.3389/fradi.2023.1141499 | Frontiers in Radiology | N/A | Genitourinary | Testicles | Yes | 6 | 2 | No | Yes | Yes | Consensus | 12.33 | QUADAS-2 |
| Fanni [30] | 2023b | 10.3390/diagnostics13162623 | Diagnostics | 3.6 | Lymphatic | Spleen | Yes | 15 | 2 | No | Yes | Yes | Consensus | 10.07 |  |
| Felfli [31] | 2023 | 10.3390/ijms241411433 | International Journal of Molecular Sciences | 5.6 | Lung |  | Yes | 28 | 2 | No | Yes | No | Consensus | 15.25 |  |
| Feng [32] | 2023a | 10.3389/fonc.2023.1194200 | Frontiers in Oncology | 4.7 | Gastrointestinal | Liver | Yes | 10 | 2 | Yes | Yes | Yes | Consensus | 14.50 | QUADAS-2 |
| Feng [33] | 2023b | 10.21037/qims-23-692 | Quantitative Imaging in Medicine and Surgery | 3.9 | Gastrointestinal | Rectum | Yes | 15 | 2 | No | Yes | Yes | Consensus | 7.73 | TRIPOD, IBSI, PROBAST |
| Fornacon-Wood [34] | 2020 | 10.1016/j.lungcan.2020.05.028 | Lung Cancer | 5.3 | Lung |  | Yes | 75 | Not specified | No | Yes | No | Not specified | 6.69 | TRIPOD |
| Gao [35] | 2022 | 10.1007/s00330-022-08922-0 | European Radiology | 5.9 | Gastrointestinal | Pancreas | Yes | 23 | 2 | Yes | Yes | Yes | Consensus | 4.78 | PROBAST |
| García-García [36] | 2022 | 10.3390/medicina58121746 | Medicina (Kaunas, Lithuania) | 2.6 | Neurology | Brain | Yes | 33 | 2 | No | Yes | Yes | Consensus | 10.67 |  |
| Granzier [37] | 2019 | 10.1016/j.ejrad.2019.108736 | European Journal of Radiology | 3.3 | Breast |  | Yes | 9 | 2 | No | Yes | Yes | Consensus | 7.78 |  |
| Gupta [38] | 2023 | 10.1016/j.wneu.2023.11.154 | World Neurosurg | 2 | Neurology | Brain | No | 14 | 2 | No | Yes | Yes | Consensus | 14.00 | IBSI, QUADAS-2 |
| HajiEsmailPoor [39] | 2023a | 10.1016/j.ejrad.2023.111129 | European Journal of Radiology | 3.3 | Endocrine | Thyroid | Yes | 25 | 2 | No | Yes | Yes | Consensus | 15.60 | QUADAS-2 |
| HajiEsmailPoor [40] | 2023b | 10.3389/fonc.2023.1185663 | Frontiers in Oncology | 4.7 | Gastrointestinal | Stomach | Yes | 15 | 2 | No | No | No | Consensus | 14.80 | QUADAS-2 |
| Harding-Theobald [41] | 2021 | 10.1111/apt.16563 | Alimentary Pharmacology & Therapeutics | 1.77 | Gastrointestinal | Liver | Yes | 54 | 2 | No | Yes | No | Consensus | 8.89 |  |
| Hou [42] | 2023 | 10.21037/qims-23-712 | Quantitative Imaging in Medicine and Surgery | 3.9 | Cardiovascular |  | No | 19 | 2 | No | Yes | Yes | Consensus | 9.89 | TRIPOD, PROBAST |
| Huang [43] | 2023a | 10.1186/s13244-023-01464-z | Insights into Imaging | 4.7 | Gynaecology | Ovaries | Yes | 57 | 2 | Yes | Yes | Yes | Average | 11.04 | QUADAS-2 |
| Huang [44] | 2023b | 10.1007/s40336-023-00593-1 | Clinical and Translational Imaging | 2.1 | Gynaecology | Cervix | Yes | 30 | 2 | No | Yes | Yes | Consensus | 8.83 | PROBAST, QUIPS |
| Janssen [45] | 2022 | 10.1097/sla.0000000000005349 | Annals of Surgery | 10.1 | Gastrointestinal | Pancreas | Yes | 23 | 2 | No | Yes | Yes | Consensus | 8.65 |  |
| Jia [46] | 2022a | 10.1016/j.ejro.2022.100438 | European Journal of Radiology Open | 2 | Lung |  | No | 13 | 2 | No | Yes | Yes | Consensus | 6.69 | QUADAS-2, CLAIM |
| Jia [47] | 2022b | 10.3389/fonc.2022.1026216 | Frontiers in Oncology | 4.7 | Gastrointestinal | Rectum | Yes | 21 | 2 | No | Yes | Yes | Consensus | 10.29 | QUADAS-2 |
| Jia [48] | 2023 | 10.1016/j.ejrad.2022.110640 | European Journal of Radiology | 3.3 | Gastrointestinal | Bowel | Yes | 29 | 2 | No | Yes | Yes | Consensus | 7.14 | QUADAS-2 |
| Jiang [49] | 2023 | 10.3389/fonc.2023.1161237 | Frontiers in Oncology | 4.7 | Gastrointestinal | Stomach | Yes | 22 | 2 | No | Yes | Yes | Consensus | 15.45 | TRIPOD, PROBAST |
| Jin [50] | 2023 | 10.1016/j.acra.2023.09.008 | Academic Radiology | 4.8 | Gastrointestinal | Liver | Yes | 49 | 2 | No | Yes | Yes | Consensus | 13.57 |  |
| Kao [51] | 2021a | 10.21873/invivo.12448 | In Vivo | 2.3 | Gastrointestinal | Oesophagus | Yes | 7 | 2 | No | Yes | Yes | Consensus | 10.14 |  |
| Kao [52] | 2021b | 10.3390/diagnostics11060991 | Diagnostics | 3.6 | Lung |  | No | 7 | 2 | No | Yes | Yes | Consensus | 13.57 |  |
| Kao [53] | 2022 | 10.1007/s11547-022-01510-8 | La Radiologia Medica | 8.9 | Lung |  | No | 8 | 2 | No | Yes | Yes | Consensus | 12.25 | QUADAS-2 |
| Kendrick [54] | 2021 | 10.3389/fonc.2021.771787 | Frontiers in Oncology | 4.7 | Genitourinary | Prostate | Yes | 17 | Not specified | No | Yes | Yes | Not specified | 7.82 |  |
| Kim [55] | 2021 | 10.1093/noajnl/vdab080 | Neuro-Oncology Advances | 3.5 | Neurology | Brain | Yes | 7 | 2 | No | Yes | Yes | Consensus | 1.29 | QUADAS-2 |
| Klontzas [56] | 2023 | 10.3390/diagnostics13122021 | Diagnostics | 3.6 | Haematology | Bone Marrow | Yes | 23 | 3 | No | Yes | No | Separate | 10.04 |  |
| Kozikowski [57] | 2022 | 10.1016/j.euf.2021.05.005 | European Urology Focus | 5.4 | Genitourinary | Bladder | Yes | 8 | 2 | No | Yes | Yes | Consensus | 14.75 | QUADAS-2 |
| Lee [58] | 2022 | 10.1007/s00330-021-08429-0 | European Radiology | 5.9 | Cardiovascular |  | No | 15 | 2 | No | No | No | Consensus | 9.9 | TRIPOD |
| Li [59] | 2022a | 10.1016/j.ejrad.2022.110243 | European Journal of Radiology | 3.3 | Gynaecology | Cervix | Yes | 12 | 2 | No | Yes | Yes | Consensus | 12.17 | QUADAS-2 |
| Li [60] | 2022b | 10.1007/s00330-022-08828-x | European Radiology | 5.9 | Neurology | Brain | Yes | 17 | 2 | Yes | Yes | Yes | Not specified | 5.53 | QUADAS-2 |
| Liang [61] | 2023 | 10.3389/fonc.2022.960944 | Frontiers in Oncology | 4.7 | Gastrointestinal | Liver | Yes | 15 | 2 | Yes | Yes | Yes | Consensus | 14.8 | QUADAS-2 |
| Lu [62] | 2023a | 10.3389/fonc.2023.1173090 | Frontiers in Oncology | 4.7 | Breast |  | Yes | 13 | 2 | No | Yes | Yes | Consensus | 20.92 |  |
| Lu [63] | 2023b | 10.1186/s12880-023-01083-6 | BMC Medical Imaging | 2.7 | Lymphatic | Thymus | Yes | 13 | 2 | No | Yes | Yes | Consensus | 9.23 | QUADAS-2 |
| Ma [64] | 2023a | 10.3389/fsurg.2022.1045295 | Frontiers in Surgery | 1.8 | Gastrointestinal | Bile Duct | Yes | 7 | 2 | Yes | Yes | Yes | Average | 16.5 | QUADAS-2 |
| Ma [65] | 2023b | 10.1016/j.ejrad.2023.111127 | European Journal of Radiology | 3.3 | Breast |  | Yes | 8 | 2 | No | Yes | Yes | Consensus | 15.13 | QUADAS-2 |
| Miccichè [66] | 2023 | 10.3389/fmed.2023.1189740 | Frontiers in Medicine | 3.9 | Gastrointestinal | Stomach | Yes | 20 | 3 | No | Yes | No | Consensus | 14.75 | TRIPOD |
| Mirza-Aghazadeh-Attari [67] | 2023 | 10.1007/s00261-023-03940-y | Abdominal Radiology | 2.4 | Gastrointestinal | Pancreas | Yes | 14 | 2 | No | Yes | Yes | Consensus | 12.29 | QUADAS-2 |
| Mombiela [68] | 2022 | 10.3390/ijms23126504 | International Journal of Molecular Sciences | 5.6 | Multiorgan |  | Yes | 45 | 5 | No | Yes | No | Consensus | 12.13 | QUADAS-2 |
| Mühlbauer [69] | 2021 | 10.3390/cancers13061348 | Cancers | 5.2 | Genitourinary | Kidney | Yes | 113 | 2 | No | Yes | No | Average | 4.88 |  |
| Nardone [70] | 2021 | 10.1007/s11547-021-01436-7 | La Radiologia Medica | 8.9 | Multiorgan |  | Yes | 43 | 2 | No | No | No | Consensus | 7.56 | QUADAS-2 |
| O'Shea [71] | 2022 | 10.1186/s13244-022-01245-0 | Insights into Imaging | 4.7 | Gastrointestinal | Oesophagus | Yes | 15 | Not specified | No | Yes | Yes | Not specified | 12.07 | TRIPOD |
| Oh [72] | 2023 | 10.7759/cureus.49015 | Cureus Journal of Medical Science | 1.2 | Breast |  | Yes | 31 | 2 | No | Yes | No | Not specified | 18.1 |  |
| Park [73] | 2022 | 10.3348/kjr.2021.0421 | Korean Journal Of Radiology | 4.8 | Neurology | Brain | Yes | 29 | 2 | No | No | No | Consensus | 3.4 | TRIPOD, IBSI |
| Park [74] | 2020a | 10.1007/s00330-019-06360-z | European Radiology | 5.9 | Multiorgan |  | Yes | 77 | 2 | No | No | No | Consensus | 9.4 | TRIPOD |
| Park [75] | 2020b | 10.1186/s12885-019-6504-5 | BMC Cancer | 3.8 | Neurology | Brain | Yes | 51 | 2 | No | No | No | Consensus | 8.92 |  |
| Patel [76] | 2023 | 10.1093/neuonc/noad028 | Neuro-Oncology | 15.9 | Neurology | Brain | Yes | 190 | 2 | No | Yes | No | Consensus | 6.72 |  |
| Pei [77] | 2023 | 10.3389/fonc.2023.1198723 | Frontiers in Oncology | 4.7 | Multiorgan |  | Yes | 22 | 2 | No | Yes | Yes | Consensus | 5.5 | NOS |
| Pesapane [78] | 2022 | 10.1016/j.currproblcancer.2022.100883 | Current Problems in Cancer | 2.6 | Breast |  | Yes | 43 | 4 | No | Yes | Yes | Consensus | 11.91 |  |
| Ponsiglione [79] | 2022 | 10.1007/s00330-021-08375-x | European Radiology | 5.9 | Cardiovascular |  | No | 53 | 2 | No | Yes | Yes | Consensus | 6.58 |  |
| Ponsiglione [80] | 2023a | 10.1007/s00330-022-09180-w | European Radiology | 5.9 | Gynaecology | Ovaries | Yes | 63 | 2 | No | Yes | Yes | Consensus | 5.59 |  |
| Ponsiglione [81] | 2023b | 10.1007/s00330-023-10427-3 | European Radiology | 5.9 | Genitourinary | Prostate | Yes | 13 | 2 | No | Yes | Yes | Consensus | 7 | QUADAS-2 |
| Ramlee [82] | 2022 | 10.3390/cancers14153656 | Cancers | 5.2 | Multiorgan |  | Yes | 27 | 2 | Yes | Yes | Yes | Average | 11.81 | QUADAS-2 |
| Ren [83] | 2022 | 10.1016/j.ejrad.2022.110504 | European Journal of Radiology | 3.3 | Gynaecology | Cervix | Yes | 8 | 2 | Yes | Yes | Yes | Consensus | 11.25 | QUADAS-2 |
| Sanduleanu [84] | 2018 | 10.1016/j.radonc.2018.03.033 | Radiotherapy and Oncology | 5.7 | Multiorgan |  | Yes | 41 | 4 | Yes | Yes | Yes | Average | 7.59 |  |
| Shahidi [85] | 2023 | 10.1007/s40520-023-02565-x | Aging Clinical and Experimental Research | 4 | Neurology | Brain | No | 13 | 3 | No | Yes | Yes | Not specified | 6.54 | QUADAS-2 |
| Shi [86] | 2021 | 10.1016/j.ejrad.2021.109956 | European Journal of Radiology | 3.3 | Lung |  | Yes | 28 | 2 | No | Yes | Yes | Consensus | 10.86 | PROBAST |
| Shi [87] | 2023 | 10.1016/j.acra.2023.05.026 | Academic Radiology | 4.8 | Lung |  | Yes | 49 | 2 | No | Yes | Yes | Consensus | 12 | PROBAST |
| Sohn [88] | 2023 | 10.1016/j.ejrad.2023.110752 | European Journal of Radiology | 3.3 | Neurology | Brain | No | 52 | 2 | No | No | No | Consensus | 3.2 | MINIMAR, TRIPOD |
| Spadarella [89] | 2021 | 10.1016/j.ejrad.2021.109744 | European Journal of Radiology | 3.3 | Head and Neck | Nasopharynx | Yes | 24 | 2 | Yes | Yes | Yes | Most experience | 7.5 |  |
| Spadarella [90] | 2022 | 10.1007/s00234-022-02959-0 | Neuroradiology | 2.8 | Head and Neck | Oropharynx | Yes | 19 | 3 | Yes | Yes | Yes | Most experience | 9.16 |  |
| Staal [91] | 2021 | 10.1016/j.clcc.2020.11.001 | Clinical Colorectal Cancer | 3.4 | Gastrointestinal | Bowel | Yes | 76 | 2 | No | Yes | Yes | Consensus | 3.54 | QUADAS-2 |
| Staal [92] | 2022 | 10.1007/s00330-022-08996-w | European Radiology | 5.9 | Gastrointestinal |  | Yes | 43 | 2 | No | Yes | Yes | Consensus | 6.11 |  |
| Stanzione [93] | 2020 | 10.1016/j.ejrad.2020.109095 | European Journal of Radiology | 3.3 | Genitourinary | Prostate | Yes | 73 | 3 | Yes | Yes | Yes | Most experience | 7.93 |  |
| Stanzione [94] | 2022a | 10.1016/j.ejrad.2022.110497 | European Journal of Radiology | 3.3 | Gynaecology | Placenta | No | 10 | 2 | No | Yes | Yes | Consensus | 6.2 |  |
| Stanzione [95] | 2022b | 10.3390/diagnostics12030578 | Diagnostics | 3.6 | Endocrine | Adrenal Glands | Yes | 25 | 2 | No | Yes | Yes | Consensus | 2.4 |  |
| Sushentsev [96] | 2022 | 10.1186/s13244-022-01199-3 | Insights into Imaging | 4.7 | Genitourinary | Prostate | Yes | 12 | 4 | No | Yes | Yes | Consensus | 10.92 | QUADAS-2, CLAIM |
| Tabnak [97] | 2023 | 10.1016/j.acra.2023.10.010 | Academic Radiology | 4.8 | Breast |  | Yes | 31 | 2 | No | Yes | Yes | Consensus | 5.9 | QUADAS-2 |
| Temperley [98] | 2023 | 10.1177/00031348231216494 | American Surgeon | 0.38 | Gastrointestinal | Anus | Yes | 9 | 2 | No | Yes | Yes | Not specified | 8 | QUADAS-2 |
| Tian [99] | 2023 | 10.3389/fonc.2023.1114983 | Frontiers in Oncology | 4.7 | Gastrointestinal | Liver | Yes | 15 | 2 | No | Yes | Yes | Consensus | 15.67 | QUADAS-2 |
| Ugga [100] | 2021 | 10.1007/s00234-021-02668-0 | Neuroradiology | 2.8 | Neurology | Brain | Yes | 23 | 3 | Yes | Yes | Yes | Most experience | 6.83 | QUADAS-2 |
| Ursprung [101] | 2020 | 10.1007/s00330-020-06666-3 | European Radiology | 5.9 | Genitourinary | Kidney | Yes | 57 | 2 | No**** | Yes | Yes | Average | 3.41 | QUADAS-2 |
| Valdora [102] | 2018 | 10.1007/s10549-018-4675-4 | Breast Cancer Research and Treatment | 3.8 | Breast |  | Yes | 17 | 2 | No | Yes | No | Consensus | 11.88 |  |
| Wakabayashi [103] | 2019 | 10.1007/s12072-019-09973-0 | Hepatology International | 6.6 | Gastrointestinal | Liver | Yes | 23 | 2 | No | Yes | No | Consensus | 8.35 |  |
| Walls [104] | 2022 | 10.1016/j.clon.2021.10.006 | Clinical Oncology | 3.4 | Lung |  | Yes | 54 | 2 | No | Yes | No | Not specified | 4.85 |  |
| Wang [105] | 2020 | 10.1007/s00330-020-06927-1 | European Radiology | 5.9 | Lymphatic |  | Yes | 45 | 2 | Yes | Yes | Yes | Average | 4.61 | QUADAS-2 |
| Wang [106] | 2021 | 10.3390/cancers13225864 | Cancers | 5.2 | Gastrointestinal | Liver | Yes | 22 | 2 | No | Yes | Yes | Consensus | 9.73 | QUADAS-2 |
| Wang [107] | 2023a | 10.1007/s11547-023-01593-x | La Radiologia medica | 8.9 | Gastrointestinal | Bowel | Yes | 12 | 2 | No | Yes | Yes | Consensus | 13.58 | QUADAS-2 |
| Wang [108] | 2023b | 10.1259/bjr.20230172 | British Journal of Radiology | 2.8 | Breast |  | Yes | 18 | 2 | Yes | Yes | Yes | Consensus | 10.17 | QUADAS-2 |
| Wang [109] | 2023c | 10.1016/j.ejrad.2023.111015 | European Journal of Radiology | 3.3 | Gastrointestinal | Liver | Yes | 11 | 2 | No | Yes | Yes | Most experience | 9.09 | QUADAS-2 |
| Wang [110] | 2023d | 10.1016/j.acra.2023.08.001 | Academic Radiology | 4.8 | Gastrointestinal | Liver | Yes | 24 | 2 | No | Yes | Yes | Consensus | 16.92 |  |
| Wei [111] | 2023 | 10.1186/s12876-023-02743-1 | BMC Gastroenterology | 2.4 | Gastrointestinal | Bowel | Yes | 68 | Not specified | No | Yes | Yes | Not specified | 10.10 |  |
| Wesdorp [112] | 2021a | 10.1007/s00259-020-05142-w | European Journal of Nuclear Medicine and Molecular Imaging | 9.1 | Gastrointestinal |  | Yes | 60 | 2 | No | Yes | Yes | Consensus | 6.68 |  |
| Wesdorp [113] | 2021b | 10.1016/j.suronc.2021.101578 | Surgical Oncology | 2.3 | Gastrointestinal | Liver | Yes | 14 | 2 | No | Yes | Yes | Consensus | 6.86 |  |
| Won [114] | 2020 | 10.3348/kjr.2020.0715 | Korean Journal of Radiology | 4.8 | Neurology | Brain | No | 26 | 2 | No | No | No | Consensus | 3.6 |  |
| Won [115] | 2021 | 10.1016/j.ejrad.2021.109673 | European Journal of Radiology | 3.3 | Neurology | Brain | Yes | 25 | 2 | No | No | No | Consensus | 5.6 | TRIPOD, IBSI |
| Won [116] | 2022 | 10.1259/bjr.20220401 | British Journal of Radiology | 2.8 | Neurology | Brain | Yes | 20 | 2 | No | No | No | Consensus | 2.8 | TRIPOD |
| Wu [117] | 2023 | 10.1007/s00330-022-09174-8 | European Radiology | 5.9 | Lung |  | Yes | 13 | 2 | No | Yes | Yes | Consensus | 13.69 | PROBAST |
| Yang [118] | 2022 | 10.3389/fonc.2022.893103 | Frontiers in Oncology | 4.7 | Head and Neck | Nasopharynx | Yes | 12 | 2 | Yes | Yes | Yes | Average | 17 | QUADAS-2 |
| Yang [119] | 2023 | 10.1097/js9.0000000000000441 | Internation Journal of Surgery | 15.3 | Gastrointestinal | Oesophagus | Yes | 46 | 2 | No | Yes | Yes | Consensus | 9.37 | QUADAS-2, TRIPOD, IBSI |
| Yuan [120] | 2023 | 10.1007/s00330-023-09414-5 | European Radiology | 5.9 | Gastrointestinal | Liver | Yes | 30 | 2 | No | Yes | Yes | Consensus | 12.97 | TRIPOD, PROBAST |
| Zhang [121] | 2022a | 10.3389/fonc.2022.799209 | Frontiers in Oncology | 4.7 | Breast |  | Yes | 13 | 2 | No | Yes | Yes | Consensus | 11.38 | QUADAS-2 |
| Zhang [122] | 2022b | 10.3389/fonc.2022.975183 | Frontiers in Oncology | 4.7 | Endocrine | Adrenal Glands | Yes | 28 | 2 | Yes | Yes | Yes | Consensus | 5.11 | QUADAS-2 |
| Zhang [123] | 2022c | 10.21037/atm-22-5986 | Annals of Translational Medicine | 3.616 | Breast |  | Yes | 43 | 2 | No | No | No | Consensus | 14.6 | PROBAST |
| Zhang [124] | 2023 | 10.3389/fonc.2023.1196053 | Frontiers in Oncology | 4.7 | Gastrointestinal | Stomach | Yes | 11 | 2 | No | Yes | Yes | Consensus | 8.91 |  |
| Zhong [125] | 2021 | 10.1007/s00330-020-07221-w | European Radiology | 5.9 | Musculoskeletal | Bone | Yes | 12 | 2 | Yes | Yes | Yes | Consensus | 6.92 | QUADAS-2 |
| Zhong [126] | 2022a | 10.1007/s00261-022-03496-3 | Abdominal Radiology | 2.4 | Gastrointestinal | Liver | Yes | 23 | Not specified | No | Yes | Yes | Not specified | 13.56 | PROBAST, TRIPOD |
| Zhong [127] | 2022b | 10.1186/s13244-022-01279-4 | Insights into Imaging | 4.7 | Gastrointestinal | Pancreas | Yes | 30 | 2 | No | Yes | Yes | Consensus | 7.27 | TRIPOD, IBSI, QUADAS-2 |
| Zhong [128] | 2022c | 10.1186/s13244-022-01277-6 | Insights into Imaging | 4.7 | Musculoskeletal | Bone | Yes | 29 | 2 | No | Yes | Yes | Consensus | 10.79 | TRIPOD, CLAIM, QUADAS-2 |
| Zhong [129] | 2023a | 10.1007/s00330-022-09060-3 | European Radiology | 5.9 | Musculoskeletal | Bone | Yes | 12 | 2 | No | Yes | Yes | Consensus | 9.08 | TRIPOD, IBSI, QUADAS-2 |
| Zhong [130] | 2023b | 10.1186/s13018-023-03863-w | Journal of Orthopaedic Surgery and Research | 2.6 | Musculoskeletal | Bone | Yes | 9 | 2 | No | Yes | Yes | Consensus | 9.33 | TRIPOD, CLAIM, QUADAS-2 |
| Zhou [131] | 2023 | 10.1016/j.clinsp.2023.100264 | Clinics | 2.7 | Gastrointestinal | Liver | Yes | 11 | 2 | No | Yes | Yes | Consensus | 13.91 | QUADAS-2, TRIPOD |
| *Additional reader(s) consulted for disagreements not included.  **Must include criteria data.  ***If final scores not directly provided, they are extracted as outlined in the methodology, if possible.  ****Multiple reader data not fully crossed.  JCR = journal citation reports, RQS = radiomics quality score, QUADAS-2 = quality assessment of diagnostic accuracy studies version 2 [132], TRIPOD = transparent reporting of a multivariable prediction model for individual prognosis or diagnosis [133], PROBAST = prediction model risk of bias assessment tool [134], IBSI = image biomarker standardisation initiative, CLAIM = checklist for artificial intelligence in medical imaging [135], QUIPS = quality in prognosis studies [136], MINIMAR = minimum information for medical artificial intelligence reporting [137], NOS = Newcastle-Ottawa scale [138]. | | | | | | | | | | | | | | | |


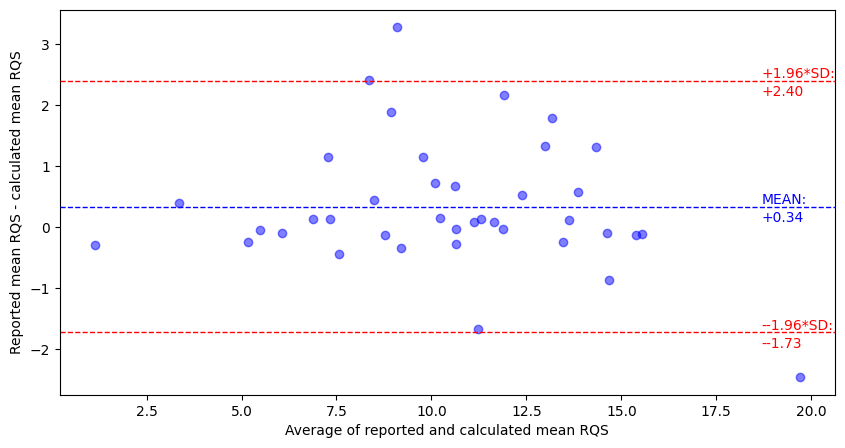


**Supplementary Fig. 3** Bland-Altman analysis of mean RQS calculated directly from criteria data provided by reviewers compared to the final scores that were reported. Mean difference (blue dashed line) and limits of agreement (red dashed line) are shown.


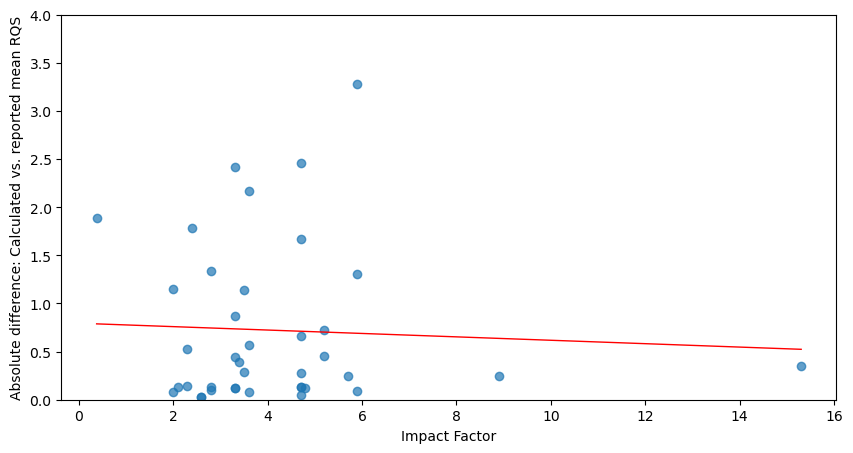


**Supplementary Fig. 4** Plot of the magnitude of errors when evaluating criteria data against the impact factor of the journal of publication of the respective review. Linear correlation (red line of best fit) was not found to be significant.


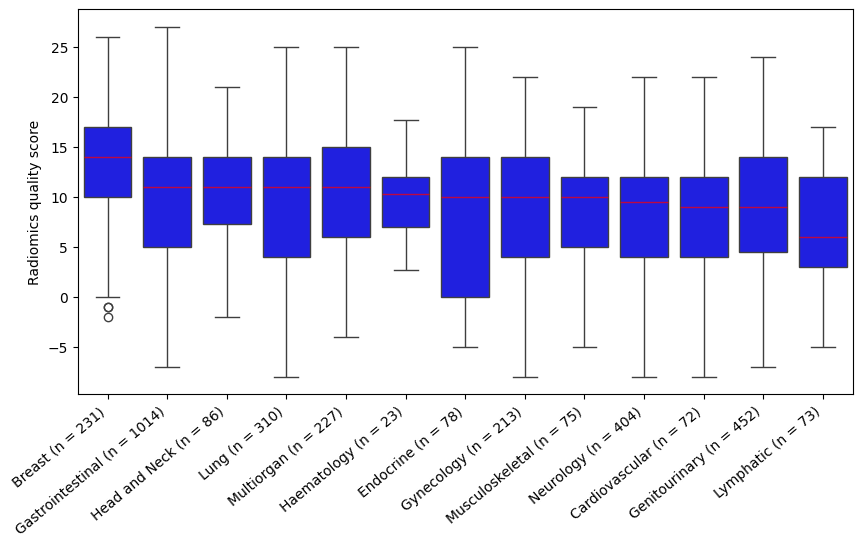


**Supplementary Fig. 5** Distribution of individually collected quality scores grouped by the system/body location that was covered by the review the respective scores were extracted from.

**
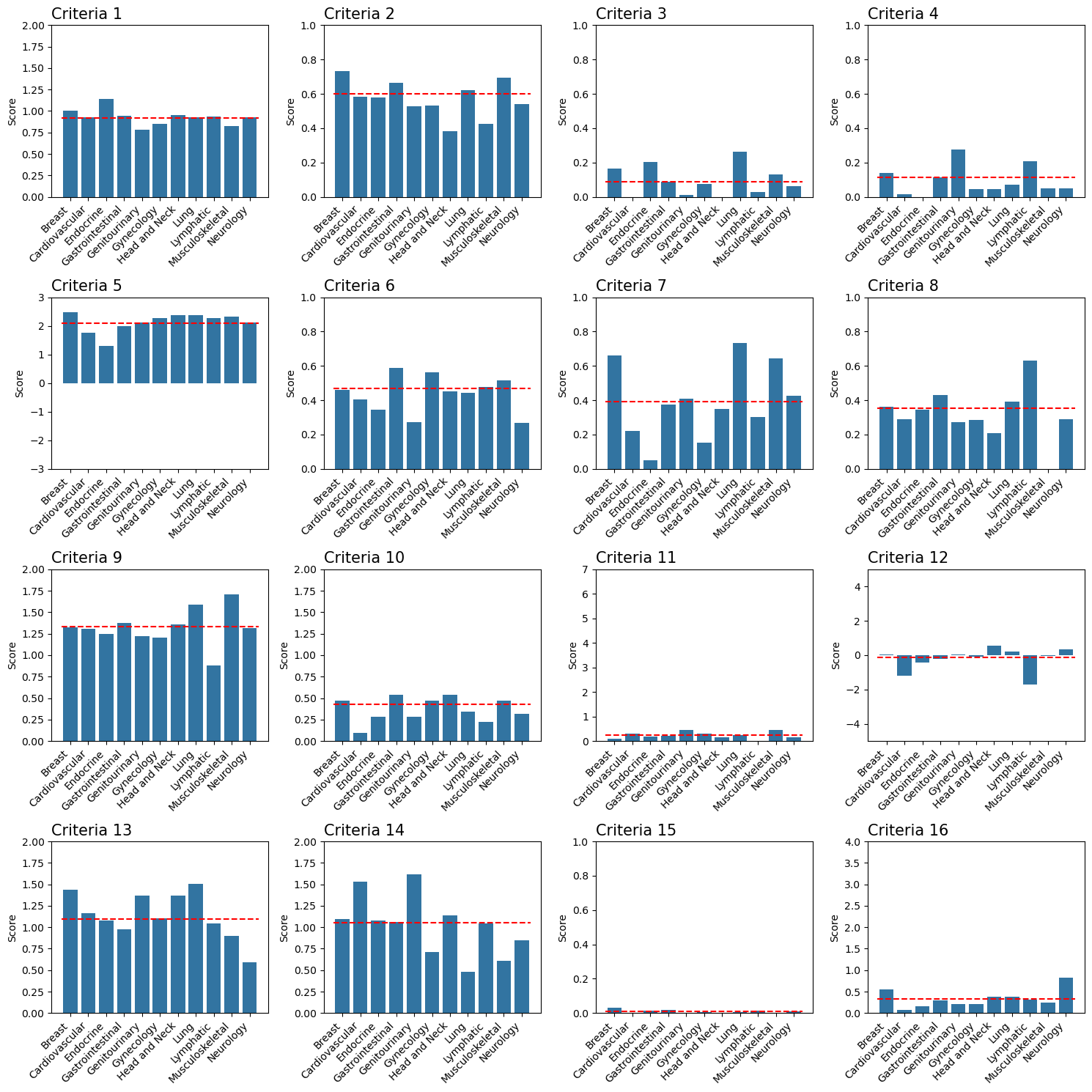
**

**Supplementary Fig. 6** A breakdown of average criteria score by the system/body location of the review the criteria were extracted from. The dotted red line represents the overall mean across reviews for comparison. Haematology is not shown as the respective review did not provide criteria scores. Multiorgan reviews are excluded.

**
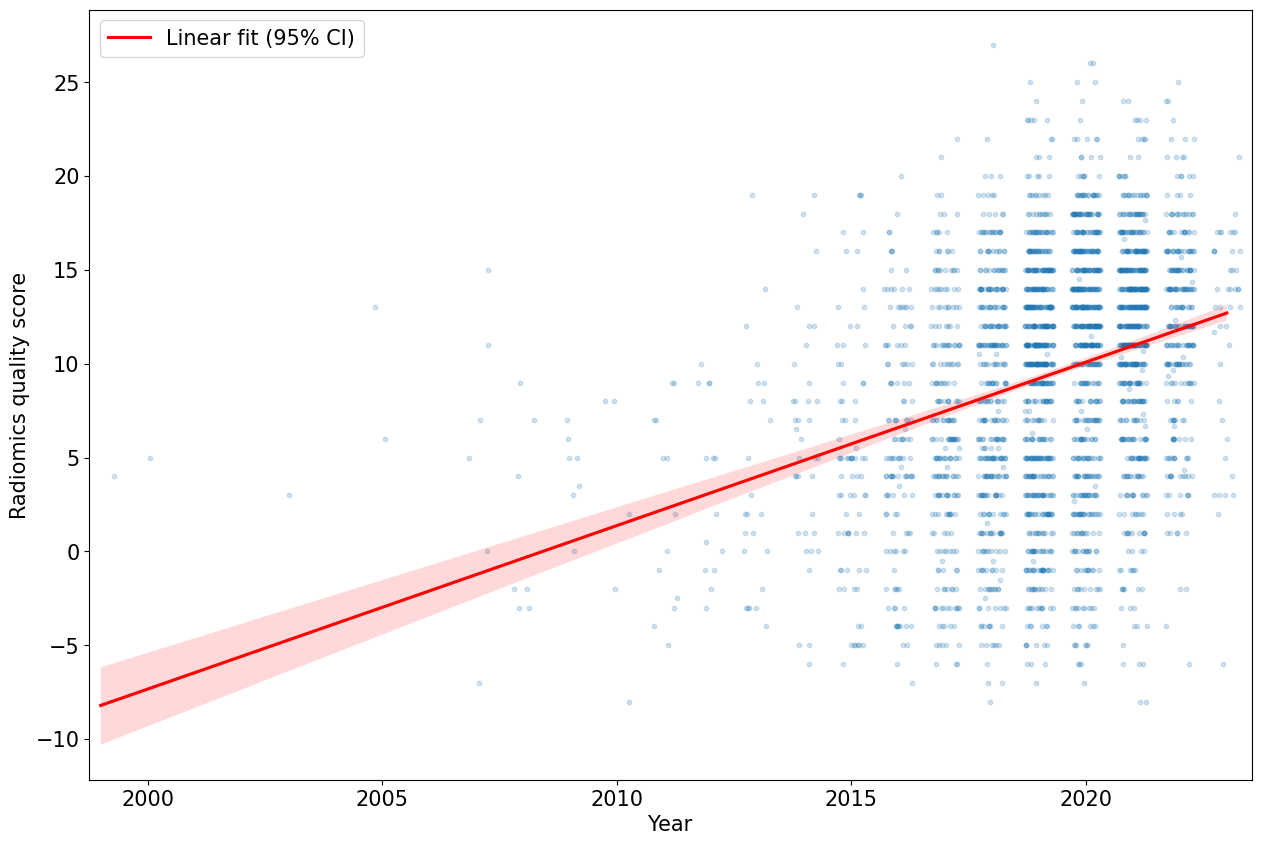
**

**Supplementary Fig. 7** Illustration of individual data points from Fig. 3 in the main manuscript. Jitter along the x-axis has been added to show density of the data. A first order line of best fit (in red) with 95% confidence interval (CI) is shown to evaluate correlation of quality scores over time.


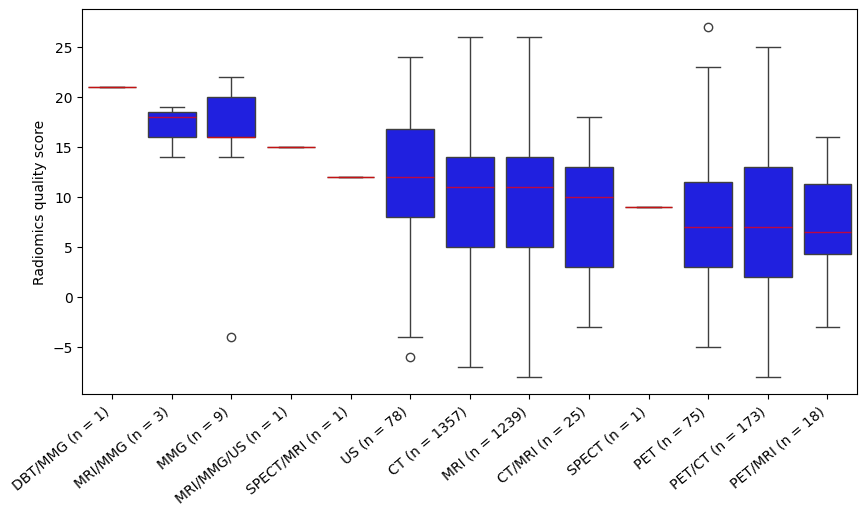


**Supplementary Fig. 8** Distribution of quality assessments grouped by the imaging modality investigated (n = 2981). Here we can see a general trend of MMG and US exhibiting higher quality scores, followed by CT and MRI, and then by PET. In Table 4 of the main text, hybrid imaging (CT/MRI, PET/MRI, PET/CT, etc) were grouped under multiple modalities when calculating the mean, although the same trends were observed. Scores of the most common modalities (CT, MRI, PET, US) were found to be significantly different.


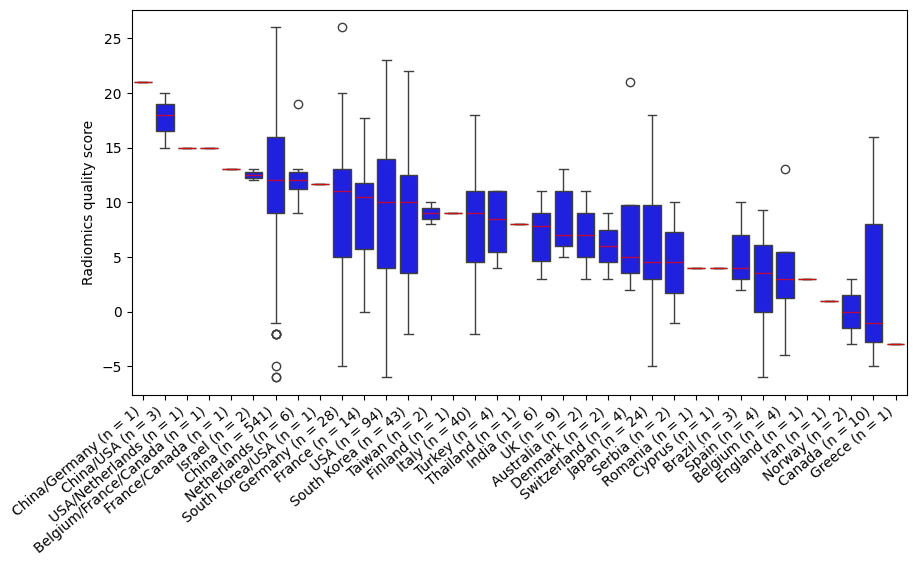


**Supplementary Fig. 9** Quality assessments grouped by the origin country of the evaluated radiomics studies are shown (n=861). One trend that can be observed here is that cooperative studies between countries (e.g., China/USA, France/Canada) tend to exhibit higher quality scores. This may be because studies between multiple countries (or institutions) are likely conducting external validation of an existing radiomics signature or model.


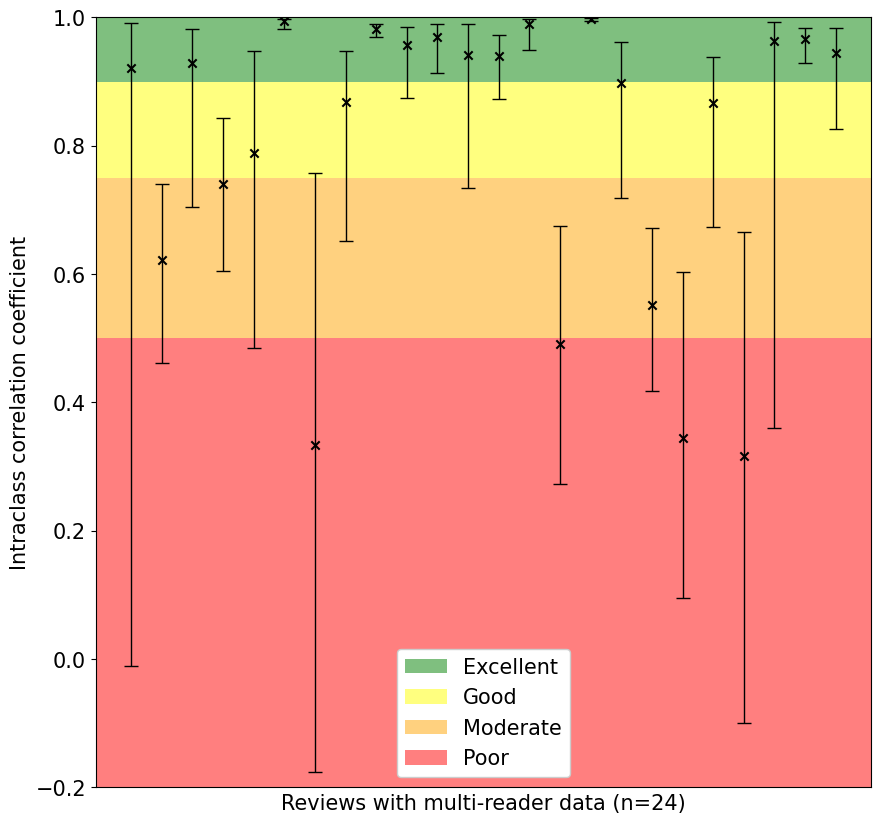


**Supplementary Fig. 10** Intraclass correlation coefficients with 95% confidence intervals (CIs) were calculated for the 24 reviews which provided data for multiple readers. The coefficients and CIs are plotted above. Interpretation of the ICC (Excellent, Good, Moderate, Poor) is based on recommendations by Koo and Li [139]**.** Table 3 in the main manuscript is derived from the lower bound of the CIs as shown.

**References**

1. Wan X, Wang W, Liu J, Tong T. Estimating the sample mean and standard deviation from the sample size, median, range and/or interquartile range. BMC Med Res Methodol. 2014;14:1-13.

2. Abbas E, Fanni SC, Bandini C, et al. Delta-radiomics in cancer immunotherapy response prediction: A systematic review. European Journal of Radiology Open. 2023;11. doi:10.1016/j.ejro.2023.100511.

3. Abdurixiti M, Nijiati M, Shen R, Ya Q, Abuduxiku N, Nijiati M. Current progress and quality of radiomic studies for predicting EGFR mutation in patients with non-small cell lung cancer using PET/CT images: a systematic review. The British Journal of Radiology. 2021;94:20201272. doi:10.1259/bjr.20201272.

4. Abunahel BM, Pontre B, Kumar H, Petrov MS. Pancreas image mining: a systematic review of radiomics. European Radiology. 2021;31:3447-67. doi:10.1007/s00330-020-07376-6.

5. Adusumilli P, Ravikumar N, Hall G, Swift S, Orsi N, Scarsbrook A. Radiomics in the evaluation of ovarian masses — a systematic review. Insights into Imaging. 2023;14. doi:10.1186/s13244-023-01500-y.

6. Albalkhi I, Bhatia A, Lösch N, Goetti R, Mankad K. Current state of radiomics in pediatric neuro-oncology practice: a systematic review. Pediatr Radiol. 2023;53:2079-91. doi:10.1007/s00247-023-05679-6.

7. Aringhieri G, Fanni SC, Febi M, Colligiani L, Cioni D, Neri E. The Role of Radiomics in Salivary Gland Imaging: A Systematic Review and Radiomics Quality Assessment. Diagnostics. 2022;12. doi:10.3390/diagnostics12123002.

8. Azadikhah A, Varghese BA, Lei X, Martin-King C, Cen SY, Duddalwar VA. Radiomics quality score in renal masses: a systematic assessment on current literature. British Journal of Radiology. 2022;95:20211211. doi:<https://dx.doi.org/10.1259/bjr.20211211>.

9. Bhandari A, Ibrahim M, Sharma C, Liong R, Gustafson S, Prior M. CT-based radiomics for differentiating renal tumours: a systematic review. Abdominal Radiology. 2021;46:2052-63. doi:10.1007/s00261-020-02832-9.

10. Bhandari AP, Liong R, Koppen J, Murthy SV, Lasocki A. Noninvasive Determination of <em>IDH</em> and 1p19q Status of Lower-grade Gliomas Using MRI Radiomics: A Systematic Review. American Journal of Neuroradiology. 2021;42:94-101. doi:10.3174/ajnr.A6875.

11. Bian JX, Wang XY, Hao W, Zhang GJ, Wang YT. The differential diagnosis value of radiomics-based machine learning in Parkinson's disease: a systematic review and meta-analysis. FRONTIERS IN AGING NEUROSCIENCE. 2023;15. doi:10.3389/fnagi.2023.1199826.

12. Boca B, Caraiani C, Telecan T, et al. MRI-Based Radiomics in Bladder Cancer: A Systematic Review and Radiomics Quality Score Assessment. DIAGNOSTICS. 2023;13. doi:10.3390/diagnostics13132300.

13. Brancato V, Cerrone M, Lavitrano M, Salvatore M, Cavaliere C. A Systematic Review of the Current Status and Quality of Radiomics for Glioma Differential Diagnosis. CANCERS. 2022;14. doi:10.3390/cancers14112731.

14. Calabrese A, Santucci D, Landi R, Beomonte Zobel B, Faiella E, de Felice C. Radiomics MRI for lymph node status prediction in breast cancer patients: the state of art. Journal of Cancer Research and Clinical Oncology. 2021;147:1587-97. doi:10.1007/s00432-021-03606-6.

15. Calimano-Ramirez LF, Virarkar MK, Hernandez M, et al. MRI-based nomograms and radiomics in presurgical prediction of extraprostatic extension in prostate cancer: a systematic review. ABDOMINAL RADIOLOGY. 2023;48:2379-400. doi:10.1007/s00261-023-03924-y.

16. Cannella R, Vernuccio F, Klontzas ME, et al. Systematic review with radiomics quality score of cholangiocarcinoma: an EuSoMII Radiomics Auditing Group Initiative. INSIGHTS INTO IMAGING. 2023;14. doi:10.1186/s13244-023-01365-1.

17. Carbonara R, Bonomo P, Di Rito A, et al. Investigation of Radiation-Induced Toxicity in Head and Neck Cancer Patients through Radiomics and Machine Learning: A Systematic Review. Journal of Oncology. 2021;2021:5566508. doi:10.1155/2021/5566508.

18. Chang S, Han K, Suh YJ, Choi BW. Quality of science and reporting for radiomics in cardiac magnetic resonance imaging studies: a systematic review. EUROPEAN RADIOLOGY. 2022;32:4361-73. doi:10.1007/s00330-022-08587-9.

19. Chen Q, Zhang L, Mo X, et al. Current status and quality of radiomic studies for predicting immunotherapy response and outcome in patients with non-small cell lung cancer: a systematic review and meta-analysis. European Journal of Nuclear Medicine and Molecular Imaging. 2021;49:345-60. doi:10.1007/s00259-021-05509-7.

20. Chen QY, Zhang L, Liu SY, et al. Radiomics in precision medicine for gastric cancer: opportunities and challenges. EUROPEAN RADIOLOGY. 2022;32:5852-68. doi:10.1007/s00330-022-08704-8.

21. Chetan MR, Gleeson FV. Radiomics in predicting treatment response in non-small-cell lung cancer: current status, challenges and future perspectives. European Radiology. 2021;31:1049-58. doi:10.1007/s00330-020-07141-9.

22. Crombé A, Fadli D, Italiano A, Saut O, Buy X, Kind M. Systematic review of sarcomas radiomics studies: Bridging the gap between concepts and clinical applications? European Journal of Radiology. 2020;132:109283. doi:<https://doi.org/10.1016/j.ejrad.2020.109283>.

23. Davey MG, Davey MS, Boland MR, Ryan ÉJ, Lowery AJ, Kerin MJ. Radiomic differentiation of breast cancer molecular subtypes using pre-operative breast imaging – A systematic review and meta-analysis. European Journal of Radiology. 2021;144:109996. doi:<https://doi.org/10.1016/j.ejrad.2021.109996>.

24. Dercle L, McGale J, Sun S, et al. Artificial intelligence and radiomics: fundamentals, applications, and challenges in immunotherapy. JOURNAL FOR IMMUNOTHERAPY OF CANCER. 2022;10. doi:10.1136/jitc-2022-005292.

25. Dragoș HM, Stan A, Pintican R, et al. MRI Radiomics and Predictive Models in Assessing Ischemic Stroke Outcome—A Systematic Review. Diagnostics. 2023;13. doi:10.3390/diagnostics13050857.

26. Du G, Zeng Y, Chen D, Zhan W, Zhan Y. Application of radiomics in precision prediction of diagnosis and treatment of gastric cancer. Jpn J Radiol. 2023;41:245-57. doi:10.1007/s11604-022-01352-4.

27. Faiella E, Santucci D, Calabrese A, et al. Artificial Intelligence in Bone Metastases: An MRI and CT Imaging Review. INTERNATIONAL JOURNAL OF ENVIRONMENTAL RESEARCH AND PUBLIC HEALTH. 2022;19. doi:10.3390/ijerph19031880.

28. Faiella E, Vaccarino F, Ragone R, et al. Can Machine Learning Models Detect and Predict Lymph Node Involvement in Prostate Cancer? A Comprehensive Systematic Review. JOURNAL OF CLINICAL MEDICINE. 2023;12. doi:10.3390/jcm12227032.

29. Fanni SC, Febi M, Colligiani L, et al. A first look into radiomics application in testicular imaging: A systematic review. Front Radiol. 2023;3:1141499. doi:10.3389/fradi.2023.1141499.

30. Fanni SC, Febi M, Francischello R, et al. Radiomics Applications in Spleen Imaging: A Systematic Review and Methodological Quality Assessment. DIAGNOSTICS. 2023;13. doi:10.3390/diagnostics13162623.

31. Felfli M, Liu Y, Zerka F, et al. Systematic Review, Meta-Analysis and Radiomics Quality Score Assessment of CT Radiomics-Based Models Predicting Tumor EGFR Mutation Status in Patients with Non-Small-Cell Lung Cancer. INTERNATIONAL JOURNAL OF MOLECULAR SCIENCES. 2023;24. doi:10.3390/ijms241411433.

32. Feng LJ, Chen QJ, Huang LJ, Long LL. Radiomics features of computed tomography and magnetic resonance imaging for predicting response to transarterial chemoembolization in hepatocellular carcinoma: a meta-analysis. FRONTIERS IN ONCOLOGY. 2023;13. doi:10.3389/fonc.2023.1194200.

33. Feng Y, Gong J, Hu T, Liu Z, Sun Y, Tong T. Radiomics for predicting survival in patients with locally advanced rectal cancer: a systematic review and meta-analysis. Quant Imaging Med Surg. 2023;13:8395-412. doi:10.21037/qims-23-692.

34. Fornacon-Wood I, Faivre-Finn C, O’Connor JPB, Price GJ. Radiomics as a personalized medicine tool in lung cancer: Separating the hope from the hype. Lung Cancer. 2020;146:197-208. doi:<https://doi.org/10.1016/j.lungcan.2020.05.028>.

35. Gao YH, Cheng SH, Zhu L, et al. A systematic review of prognosis predictive role of radiomics in pancreatic cancer: heterogeneity markers or statistical tricks? EUROPEAN RADIOLOGY. 2022;32:8443-52. doi:10.1007/s00330-022-08922-0.

36. García-García S, García-Galindo M, Arrese I, Sarabia R, Cepeda S. Current Evidence, Limitations and Future Challenges of Survival Prediction for Glioblastoma Based on Advanced Noninvasive Methods: A Narrative Review. MEDICINA-LITHUANIA. 2022;58. doi:10.3390/medicina58121746.

37. Granzier RWY, van Nijnatten TJA, Woodruff HC, Smidt ML, Lobbes MBI. Exploring breast cancer response prediction to neoadjuvant systemic therapy using MRI-based radiomics: A systematic review. European Journal of Radiology. 2019;121:108736. doi:<https://doi.org/10.1016/j.ejrad.2019.108736>.

38. Gupta R, Bilgin C, Jabal MS, et al. Quality Assessment of Radiomics Studies on Functional Outcomes Following Acute Ischemic Stroke - A Systematic Review. World Neurosurg. 2023. doi:10.1016/j.wneu.2023.11.154.

39. HajiEsmailPoor Z, Kargar Z, Tabnak P. Radiomics diagnostic performance in predicting lymph node metastasis of papillary thyroid carcinoma: A systematic review and meta-analysis. EUROPEAN JOURNAL OF RADIOLOGY. 2023;168. doi:10.1016/j.ejrad.2023.111129.

40. HajiEsmailpoor Z, Tabnak P, Baradaran B, Pashazadeh F, Aghebati-Maleki L. Diagnostic performance of CT scan-based radiomics for prediction of lymph node metastasis in gastric cancer: a systematic review and meta-analysis. FRONTIERS IN ONCOLOGY. 2023;13. doi:10.3389/fonc.2023.1185663.

41. Harding-Theobald E, Louissaint J, Maraj B, et al. Systematic review: radiomics for the diagnosis and prognosis of hepatocellular carcinoma. Alimentary Pharmacology & Therapeutics. 2021;54:890-901. doi:<https://doi.org/10.1111/apt.16563>.

42. Hou C, Li S, Zheng S, et al. Quality assessment of radiomics models in carotid plaque: a systematic review. QUANTITATIVE IMAGING IN MEDICINE AND SURGERY. 2023. doi:10.21037/qims-23-712.

43. Huang ML, Ren J, Jin ZY, et al. A systematic review and meta-analysis of CT and MRI radiomics in ovarian cancer: methodological issues and clinical utility. INSIGHTS INTO IMAGING. 2023;14. doi:10.1186/s13244-023-01464-z.

44. Huang LZ, Li L, Huang XQ, et al. A systematic review of radiomics for predicting treatment response and survival in locally advanced cervical cancer: positive results or optimistic illusions? CLINICAL AND TRANSLATIONAL IMAGING. 2023. doi:10.1007/s40336-023-00593-1.

45. Janssen BV, Verhoef S, Wesdorp NJ, et al. Imaging-based Machine-learning Models to Predict Clinical Outcomes and Identify Biomarkers in Pancreatic Cancer: A Scoping Review. Annals of Surgery. 2022;275:560-7. doi:10.1097/sla.0000000000005349.

46. Jia LL, Zhao JX, Pan NN, et al. Artificial intelligence model on chest imaging to diagnose COVID-19 and other pneumonias: A systematic review and meta-analysis. EUROPEAN JOURNAL OF RADIOLOGY OPEN. 2022;9. doi:10.1016/j.ejro.2022.100438.

47. Jia LL, Zheng QY, Tian JH, et al. Artificial intelligence with magnetic resonance imaging for prediction of pathological complete response to neoadjuvant chemoradiotherapy in rectal cancer: A systematic review and meta-analysis. FRONTIERS IN ONCOLOGY. 2022;12. doi:10.3389/fonc.2022.1026216.

48. Jia LL, Zhao JX, Zhao LP, Tian JH, Huang G. Current status and quality of radiomic studies for predicting KRAS mutations in colorectal cancer patients: A systematic review and meta-analysis. EUROPEAN JOURNAL OF RADIOLOGY. 2023;158. doi:10.1016/j.ejrad.2022.110640.

49. Jiang T, Zhao Z, Liu X, et al. Methodological quality of radiomic-based prognostic studies in gastric cancer: a cross-sectional study. Frontiers in Oncology. 2023;13. doi:10.3389/fonc.2023.1161237.

50. Jin J, Jiang Y, Zhao YL, Huang PT. Radiomics-based Machine Learning to Predict the Recurrence of Hepatocellular Carcinoma: A Systematic Review and Meta-analysis. Acad Radiol. 2023. doi:10.1016/j.acra.2023.09.008.

51. KAO Y-S, HSU Y. A Meta-Analysis for Using Radiomics to Predict Complete Pathological Response in Esophageal Cancer Patients Receiving Neoadjuvant Chemoradiation. In Vivo. 2021;35:1857-63. doi:10.21873/invivo.12448.

52. Kao Y-S, Lin K-T. A Meta-Analysis of Computerized Tomography-Based Radiomics for the Diagnosis of COVID-19 and Viral Pneumonia. Diagnostics. 2021;11:991.

53. Kao YS, Lin KT. A meta-analysis of the diagnostic test accuracy of CT-based radiomics for the prediction of COVID-19 severity. RADIOLOGIA MEDICA. 2022;127:754-62. doi:10.1007/s11547-022-01510-8.

54. Kendrick J, Francis R, Hassan GM, et al. Radiomics for Identification and Prediction in Metastatic Prostate Cancer: A Review of Studies. Frontiers in Oncology. 2021;11. doi:10.3389/fonc.2021.771787.

55. Kim HY, Cho SJ, Sunwoo L, et al. Classification of true progression after radiotherapy of brain metastasis on MRI using artificial intelligence: a systematic review and meta-analysis. Neuro-Oncology Advances. 2021;3. doi:10.1093/noajnl/vdab080.

56. Klontzas ME, Triantafyllou M, Leventis D, et al. Radiomics Analysis for Multiple Myeloma: A Systematic Review with Radiomics Quality Scoring. DIAGNOSTICS. 2023;13. doi:10.3390/diagnostics13122021.

57. Kozikowski M, Suarez-Ibarrola R, Osiecki R, et al. Role of Radiomics in the Prediction of Muscle-invasive Bladder Cancer: A Systematic Review and Meta-analysis. European Urology Focus. 2022;8:728-38. doi:<https://doi.org/10.1016/j.euf.2021.05.005>.

58. Lee S, Han K, Suh YJ. Quality assessment of radiomics research in cardiac CT: a systematic review. EUROPEAN RADIOLOGY. 2022;32:3458-68. doi:10.1007/s00330-021-08429-0.

59. Li LC, Zhang J, Zhe X, et al. A meta-analysis of MRI-based radiomic features for predicting lymph node metastasis in patients with cervical cancer. EUROPEAN JOURNAL OF RADIOLOGY. 2022;151. doi:10.1016/j.ejrad.2022.110243.

60. Li YZ, Liu YJ, Liang YY, et al. Radiomics can differentiate high-grade glioma from brain metastasis: a systematic review and meta-analysis. EUROPEAN RADIOLOGY. 2022;32:8039-51. doi:10.1007/s00330-022-08828-x.

61. Liang G, Yu W, Liu SQ, et al. The diagnostic performance of radiomics-based MRI in predicting microvascular invasion in hepatocellular carcinoma: A meta-analysis. FRONTIERS IN ONCOLOGY. 2023;12. doi:10.3389/fonc.2022.960944.

62. Lu DM, Yan YK, Jiang M, et al. Predictive value of radiomics-based machine learning for the disease-free survival in breast cancer: a systematic review and meta-analysis. FRONTIERS IN ONCOLOGY. 2023;13. doi:10.3389/fonc.2023.1173090.

63. Lu XF, Zhu TY. Diagnostic performance of radiomics model for preoperative risk categorization in thymic epithelial tumors: a systematic review and meta-analysis. BMC MEDICAL IMAGING. 2023;23. doi:10.1186/s12880-023-01083-6.

64. Ma YH, Lin YY, Lu JY, et al. A meta-analysis of based radiomics for predicting lymph node metastasis in patients with biliary tract cancers. FRONTIERS IN SURGERY. 2023;9. doi:10.3389/fsurg.2022.1045295.

65. Ma Q, Li Z, Li W, et al. MRI radiomics for the preoperative evaluation of lymphovascular invasion in breast cancer: A meta-analysis. Eur J Radiol. 2023;168:111127. doi:10.1016/j.ejrad.2023.111127.

66. Miccichè F, Rizzo G, Casà C, et al. Role of radiomics in predicting lymph node metastasis in gastric cancer: a systematic review. Front Med. 2023;10. doi:10.3389/fmed.2023.1189740.

67. Mirza-Aghazadeh-Attari M, Madani SP, Shahbazian H, et al. Predictive role of radiomics features extracted from preoperative cross-sectional imaging of pancreatic ductal adenocarcinoma in detecting lymph node metastasis: a systemic review and meta-analysis. ABDOMINAL RADIOLOGY. 2023;48:2570-84. doi:10.1007/s00261-023-03940-y.

68. Mombiela RM, Arildskov AR, Bruun FJ, et al. What Genetics Can Do for Oncological Imaging: A Systematic Review of the Genetic Validation Data Used in Radiomics Studies. INTERNATIONAL JOURNAL OF MOLECULAR SCIENCES. 2022;23. doi:10.3390/ijms23126504.

69. Mühlbauer J, Egen L, Kowalewski K-F, et al. Radiomics in Renal Cell Carcinoma—A Systematic Review and Meta-Analysis. Cancers. 2021;13:1348.

70. Nardone V, Reginelli A, Grassi R, et al. Delta radiomics: a systematic review. La radiologia medica. 2021;126:1571-83. doi:10.1007/s11547-021-01436-7.

71. O'Shea RJ, Rookyard C, Withey S, Cook GJR, Tsoka S, Goh V. Radiomic assessment of oesophageal adenocarcinoma: a critical review of 18F-FDG PET/CT, PET/MRI and CT. INSIGHTS INTO IMAGING. 2022;13. doi:10.1186/s13244-022-01245-0.

72. Oh KE, Vasandani N, Anwar A. Radiomics to Differentiate Malignant and Benign Breast Lesions: A Systematic Review and Diagnostic Test Accuracy Meta-Analysis. CUREUS JOURNAL OF MEDICAL SCIENCE. 2023;15. doi:10.7759/cureus.49015.

73. Park CJ, Park YW, Ahn SS, et al. Quality of Radiomics Research on Brain Metastasis: A Roadmap to Promote Clinical Translation. KOREAN JOURNAL OF RADIOLOGY. 2022;23:77-88. doi:10.3348/kjr.2021.0421.

74. Park JE, Kim D, Kim HS, et al. Quality of science and reporting of radiomics in oncologic studies: room for improvement according to radiomics quality score and TRIPOD statement. European Radiology. 2020;30:523-36. doi:10.1007/s00330-019-06360-z.

75. Park JE, Kim HS, Kim D, et al. A systematic review reporting quality of radiomics research in neuro-oncology: toward clinical utility and quality improvement using high-dimensional imaging features. BMC Cancer. 2020;20:29. doi:10.1186/s12885-019-6504-5.

76. Patel RV, Yao S, Huang RY, Bi WL. Application of radiomics to meningiomas: A systematic review. NEURO-ONCOLOGY. 2023;25:1166-76. doi:10.1093/neuonc/noad028.

77. Pei XY, Xie Y, Liu YX, et al. Imaging-based adipose biomarkers for predicting clinical outcomes of cancer patients treated with immune checkpoint inhibitors: a systematic review. FRONTIERS IN ONCOLOGY. 2023;13. doi:10.3389/fonc.2023.1198723.

78. Pesapane F, Agazzi GM, Rotili A, et al. Prediction of the Pathological Response to Neoadjuvant Chemotherapy in Breast Cancer Patients With MRI-Radiomics: A Systematic Review and Meta-analysis. CURRENT PROBLEMS IN CANCER. 2022;46. doi:10.1016/j.currproblcancer.2022.100883.

79. Ponsiglione A, Stanzione A, Cuocolo R, et al. Cardiac CT and MRI radiomics: systematic review of the literature and radiomics quality score assessment. European Radiology. 2022;32:2629-38. doi:10.1007/s00330-021-08375-x.

80. Ponsiglione A, Stanzione A, Spadarella G, et al. Ovarian imaging radiomics quality score assessment: an EuSoMII radiomics auditing group initiative. EUROPEAN RADIOLOGY. 2023;33:2239-47. doi:10.1007/s00330-022-09180-w.

81. Ponsiglione A, Gambardella M, Stanzione A, et al. Radiomics for the identification of extraprostatic extension with prostate MRI: a systematic review and meta-analysis. EUROPEAN RADIOLOGY. 2023. doi:10.1007/s00330-023-10427-3.

82. Ramlee S, Hulse D, Bernatowicz K, Pérez-López R, Sala E, Aloj L. Radiomic Signatures Associated with CD8(+) Tumour-Infiltrating Lymphocytes: A Systematic Review and Quality Assessment Study. Cancers (Basel). 2022;14. doi:10.3390/cancers14153656.

83. Ren J, Li Y, Liu XY, et al. Diagnostic performance of ADC values and MRI-based radiomics analysis for detecting lymph node metastasis in patients with cervical cancer: A systematic review and meta-analysis. EUROPEAN JOURNAL OF RADIOLOGY. 2022;156. doi:10.1016/j.ejrad.2022.110504.

84. Sanduleanu S, Woodruff HC, De Jong EE, et al. Tracking tumor biology with radiomics: a systematic review utilizing a radiomics quality score. Radiother Oncol. 2018;127:349-60.

85. Shahidi R, Baradaran M, Asgarzadeh A, et al. Diagnostic performance of MRI radiomics for classification of Alzheimer's disease, mild cognitive impairment, and normal subjects: a systematic review and meta-analysis. Aging Clin Exp Res. 2023;35:2333-48. doi:10.1007/s40520-023-02565-x.

86. Shi L, Zhao J, Peng X, Wang Y, Liu L, Sheng M. CT-based radiomics for differentiating invasive adenocarcinomas from indolent lung adenocarcinomas appearing as ground-glass nodules: Asystematic review. European Journal of Radiology. 2021;144:109956. doi:<https://doi.org/10.1016/j.ejrad.2021.109956>.

87. Shi LL, Sheng MH, Wei ZC, Liu L, Zhao JL. CT-Based Radiomics Predicts the Malignancy of Pulmonary Nodules: A Systematic Review and Meta-Analysis. ACADEMIC RADIOLOGY. 2023;30:3064-75. doi:10.1016/j.acra.2023.05.026.

88. Sohn B, Won SY. Quality assessment of stroke radiomics studies: Promoting clinical application. EUROPEAN JOURNAL OF RADIOLOGY. 2023;161. doi:10.1016/j.ejrad.2023.110752.

89. Spadarella G, Calareso G, Garanzini E, Ugga L, Cuocolo A, Cuocolo R. MRI based radiomics in nasopharyngeal cancer: Systematic review and perspectives using radiomic quality score (RQS) assessment. European Journal of Radiology. 2021;140:109744. doi:<https://doi.org/10.1016/j.ejrad.2021.109744>.

90. Spadarella G, Ugga L, Calareso G, Villa R, D'Aniello S, Cuocolo R. The impact of radiomics for human papillomavirus status prediction in oropharyngeal cancer: systematic review and radiomics quality score assessment. NEURORADIOLOGY. 2022;64:1639-47. doi:10.1007/s00234-022-02959-0.

91. Staal FCR, van der Reijd DJ, Taghavi M, Lambregts DMJ, Beets-Tan RGH, Maas M. Radiomics for the Prediction of Treatment Outcome and Survival in Patients With Colorectal Cancer: A Systematic Review. Clinical Colorectal Cancer. 2021;20:52-71. doi:<https://doi.org/10.1016/j.clcc.2020.11.001>.

92. Staal FCR, Aalbersberg EA, van der Velden D, et al. GEP-NET radiomics: a systematic review and radiomics quality score assessment. EUROPEAN RADIOLOGY. 2022;32:7278-94. doi:10.1007/s00330-022-08996-w.

93. Stanzione A, Gambardella M, Cuocolo R, Ponsiglione A, Romeo V, Imbriaco M. Prostate MRI radiomics: A systematic review and radiomic quality score assessment. European Journal of Radiology. 2020;129:109095. doi:<https://doi.org/10.1016/j.ejrad.2020.109095>.

94. Stanzione A, Verde F, Cuocolo R, et al. Placenta Accreta Spectrum Disorders and Radiomics: Systematic review and quality appraisal. EUROPEAN JOURNAL OF RADIOLOGY. 2022;155. doi:10.1016/j.ejrad.2022.110497.

95. Stanzione A, Galatola R, Cuocolo R, et al. Radiomics in Cross-Sectional Adrenal Imaging: A Systematic Review and Quality Assessment Study. DIAGNOSTICS. 2022;12. doi:10.3390/diagnostics12030578.

96. Sushentsev N, Da Silva NM, Yeung M, et al. Comparative performance of fully-automated and semi-automated artificial intelligence methods for the detection of clinically significant prostate cancer on MRI: a systematic review. INSIGHTS INTO IMAGING. 2022;13. doi:10.1186/s13244-022-01199-3.

97. Tabnak P, HajiEsmailPoor Z, Baradaran B, Pashazadeh F, Aghebati Maleki L. MRI-Based Radiomics Methods for Predicting Ki-67 Expression in Breast Cancer: A Systematic Review and Meta-analysis. Acad Radiol. 2023. doi:10.1016/j.acra.2023.10.010.

98. Temperley HC, O'Sullivan NJ, Waters C, et al. Radiomics; Contemporary Applications in the Management of Anal Cancer; A Systematic Review. AMERICAN SURGEON. 2023. doi:10.1177/00031348231216494.

99. Tian H, Xie Y, Wang ZQ. Radiomics for preoperative prediction of early recurrence in hepatocellular carcinoma: a meta-analysis. FRONTIERS IN ONCOLOGY. 2023;13. doi:10.3389/fonc.2023.1114983.

100. Ugga L, Perillo T, Cuocolo R, et al. Meningioma MRI radiomics and machine learning: systematic review, quality score assessment, and meta-analysis. Neuroradiology. 2021;63:1293-304. doi:10.1007/s00234-021-02668-0.

101. Ursprung S, Beer L, Bruining A, et al. Radiomics of computed tomography and magnetic resonance imaging in renal cell carcinoma—a systematic review and meta-analysis. European Radiology. 2020;30:3558-66. doi:10.1007/s00330-020-06666-3.

102. Valdora F, Houssami N, Rossi F, Calabrese M, Tagliafico AS. Rapid review: radiomics and breast cancer. Breast Cancer Research and Treatment. 2018;169:217-29. doi:10.1007/s10549-018-4675-4.

103. Wakabayashi T, Ouhmich F, Gonzalez-Cabrera C, et al. Radiomics in hepatocellular carcinoma: a quantitative review. Hepatology International. 2019;13:546-59. doi:10.1007/s12072-019-09973-0.

104. Walls GM, Osman SOS, Brown KH, et al. Radiomics for Predicting Lung Cancer Outcomes Following Radiotherapy: A Systematic Review. Clinical Oncology. 2022;34:e107-e22. doi:<https://doi.org/10.1016/j.clon.2021.10.006>.

105. Wang H, Zhou Y, Li L, Hou W, Ma X, Tian R. Current status and quality of radiomics studies in lymphoma: a systematic review. European Radiology. 2020;30:6228-40. doi:10.1007/s00330-020-06927-1.

106. Wang Q, Li C, Zhang J, et al. Radiomics Models for Predicting Microvascular Invasion in Hepatocellular Carcinoma: A Systematic Review and Radiomics Quality Score Assessment. Cancers. 2021;13:5864.

107. Wang Q, Xu JH, Wang AR, et al. Systematic review of machine learning-based radiomics approach for predicting microsatellite instability status in colorectal cancer. RADIOLOGIA MEDICA. 2023;128:136-48. doi:10.1007/s11547-023-01593-x.

108. Wang M, Mei T, Gong YL. The quality and clinical translation of radiomics studies based on MRI for predicting Ki-67 levels in patients with breast cancer. BRITISH JOURNAL OF RADIOLOGY. 2023;96. doi:10.1259/bjr.20230172.

109. Wang Q, Wang AR, Wu XY, et al. Radiomics models for preoperative prediction of the histopathological grade of hepatocellular carcinoma: A systematic review and radiomics quality score assessment. EUROPEAN JOURNAL OF RADIOLOGY. 2023;166. doi:10.1016/j.ejrad.2023.111015.

110. Wang Y, Li M, Zhang Z, Gao M, Zhao L. Application of Radiomics in the Efficacy Evaluation of Transarterial Chemoembolization for Hepatocellular Carcinoma: A Systematic Review and Meta-analysis. Acad Radiol. 2023. doi:10.1016/j.acra.2023.08.001.

111. Wei X, Feng X, Liu Y, Feng G, Du Y. Repeatability of radiomics studies in colorectal cancer: a systematic review. BMC Gastroenterology. 2023;23:125. doi:<https://dx.doi.org/10.1186/s12876-023-02743-1>.

112. Wesdorp NJ, Hellingman T, Jansma EP, et al. Advanced analytics and artificial intelligence in gastrointestinal cancer: a systematic review of radiomics predicting response to treatment. European Journal of Nuclear Medicine and Molecular Imaging. 2021;48:1785-94. doi:10.1007/s00259-020-05142-w.

113. Wesdorp NJ, van Goor VJ, Kemna R, et al. Advanced image analytics predicting clinical outcomes in patients with colorectal liver metastases: A systematic review of the literature. Surgical Oncology. 2021;38:101578. doi:<https://doi.org/10.1016/j.suronc.2021.101578>.

114. Won SY, Park YW, Park M, Ahn SS, Kim J, Lee S-K. Quality Reporting of Radiomics Analysis in Mild Cognitive Impairment and Alzheimer's Disease: A Roadmap for Moving Forward. Korean J Radiol. 2020;21:1345-54.

115. Won SY, Park YW, Ahn SS, et al. Quality assessment of meningioma radiomics studies: Bridging the gap between exploratory research and clinical applications. European Journal of Radiology. 2021;138:109673. doi:<https://doi.org/10.1016/j.ejrad.2021.109673>.

116. Won SY, Lee NR, Park YW, et al. Quality reporting of radiomics analysis in pituitary adenomas: promoting clinical translation. BRITISH JOURNAL OF RADIOLOGY. 2022;95. doi:10.1259/bjr.20220401.

117. Wu LY, Lou XJ, Kong N, Xu MS, Gao C. Can quantitative peritumoral CT radiomics features predict the prognosis of patients with non-small cell lung cancer? A systematic review. EUROPEAN RADIOLOGY. 2023;33:2105-17. doi:10.1007/s00330-022-09174-8.

118. Yang C, Jiang ZK, Cheng TT, et al. Radiomics for Predicting Response of Neoadjuvant Chemotherapy in Nasopharyngeal Carcinoma: A Systematic Review and Meta-Analysis. FRONTIERS IN ONCOLOGY. 2022;12. doi:10.3389/fonc.2022.893103.

119. Yang Z, Gong J, Li J, Sun HF, Pan YL, Zhao LA. The gap before real clinical application of imaging-based machine-learning and radiomic models for chemoradiation outcome prediction in esophageal cancer: a systematic review and meta-analysis. INTERNATIONAL JOURNAL OF SURGERY. 2023;109:2451-66. doi:10.1097/JS9.0000000000000441.

120. Yuan EY, Chen YT, Song B. Quality of radiomics for predicting microvascular invasion in hepatocellular carcinoma: a systematic review. EUROPEAN RADIOLOGY. 2023;33:3467-77. doi:10.1007/s00330-023-09414-5.

121. Zhang J, Li LC, Zhe X, et al. The Diagnostic Performance of Machine Learning-Based Radiomics of DCE-MRI in Predicting Axillary Lymph Node Metastasis in Breast Cancer: A Meta-Analysis. FRONTIERS IN ONCOLOGY. 2022;12. doi:10.3389/fonc.2022.799209.

122. Zhang H, Lei HQ, Pang J. Diagnostic performance of radiomics in adrenal masses: A systematic review and meta-analysis. FRONTIERS IN ONCOLOGY. 2022;12. doi:10.3389/fonc.2022.975183.

123. Zhang YW, Li GF, Bian WQ, et al. Value of genomics- and radiomics-based machine learning models in the identification of breast cancer molecular subtypes: a systematic review and meta-analysis. ANNALS OF TRANSLATIONAL MEDICINE. 2022;10. doi:10.21037/atm-22-5986.

124. Zhang F, Wu GX, Chen N, Li RY. The predictive value of radiomics-based machine learning for peritoneal metastasis in gastric cancer patients: a systematic review and meta-analysis. FRONTIERS IN ONCOLOGY. 2023;13. doi:10.3389/fonc.2023.1196053.

125. Zhong J, Hu Y, Si L, et al. A systematic review of radiomics in osteosarcoma: utilizing radiomics quality score as a tool promoting clinical translation. European Radiology. 2021;31:1526-35. doi:10.1007/s00330-020-07221-w.

126. Zhong X, Long HY, Su LY, et al. Radiomics models for preoperative prediction of microvascular invasion in hepatocellular carcinoma: a systematic review and meta-analysis. ABDOMINAL RADIOLOGY. 2022;47:2071-88. doi:10.1007/s00261-022-03496-3.

127. Zhong JY, Hu YF, Xing Y, et al. A systematic review of radiomics in pancreatitis: applying the evidence level rating tool for promoting clinical transferability. INSIGHTS INTO IMAGING. 2022;13. doi:10.1186/s13244-022-01279-4.

128. Zhong JY, Hu YF, Zhang GC, et al. An updated systematic review of radiomics in osteosarcoma: utilizing CLAIM to adapt the increasing trend of deep learning application in radiomics. INSIGHTS INTO IMAGING. 2022;13. doi:10.1186/s13244-022-01277-6.

129. Zhong JY, Hu YF, Ge X, et al. A systematic review of radiomics in chondrosarcoma: assessment of study quality and clinical value needs handy tools. EUROPEAN RADIOLOGY. 2023;33:1433-44. doi:10.1007/s00330-022-09060-3.

130. Zhong JY, Xing Y, Zhang GC, et al. A systematic review of radiomics in giant cell tumor of bone (GCTB): the potential of analysis on individual radiomics feature for identifying genuine promising imaging biomarkers. JOURNAL OF ORTHOPAEDIC SURGERY AND RESEARCH. 2023;18. doi:10.1186/s13018-023-03863-w.

131. Zhou HY, Cheng JM, Chen TW, et al. CT radiomics for prediction of microvascular invasion in hepatocellular carcinoma: A systematic review and meta-analysis. CLINICS. 2023;78. doi:10.1016/j.clinsp.2023.100264.

132. Whiting PF, Rutjes AW, Westwood ME, et al. QUADAS-2: a revised tool for the quality assessment of diagnostic accuracy studies. Ann Intern Med. 2011;155:529-36.

133. Collins GS, Reitsma JB, Altman DG, Moons KG. Transparent Reporting of a multivariable prediction model for Individual Prognosis or Diagnosis (TRIPOD): the TRIPOD statement. Ann Intern Med. 2015;162:55-63. doi:10.7326/M14-0697.

134. Wolff RF, Moons KG, Riley RD, et al. PROBAST: a tool to assess the risk of bias and applicability of prediction model studies. Ann Intern Med. 2019;170:51-8.

135. Mongan J, Moy L, Charles E. Kahn J. Checklist for Artificial Intelligence in Medical Imaging (CLAIM): A Guide for Authors and Reviewers. Radiol Artif Intell. 2020;2:e200029. doi:10.1148/ryai.2020200029.

136. Hayden JA, van der Windt DA, Cartwright JL, Côté P, Bombardier C. Assessing bias in studies of prognostic factors. Ann Intern Med. 2013;158:280-6.

137. Hernandez-Boussard T, Bozkurt S, Ioannidis JP, Shah NH. MINIMAR (MINimum Information for Medical AI Reporting): Developing reporting standards for artificial intelligence in health care. J Am Med Inform Assoc. 2020;27:2011-5.

138. Lo CK-L, Mertz D, Loeb M. Newcastle-Ottawa Scale: comparing reviewers’ to authors’ assessments. BMC Med Res Methodol. 2014;14:1-5.

139. Koo TK, Li MY. A guideline of selecting and reporting intraclass correlation coefficients for reliability research. J Chiropr Med. 2016;15:155-63. doi:10.1016/j.jcm.2016.02.012.
